# DNA Origami Tension Sensors (DOTS) for Single-Molecule Force Measurements at Fluid Intermembrane Junctions

**DOI:** 10.1021/acs.nanolett.5c02130

**Published:** 2025-08-25

**Authors:** Sarah Al Abdullatif, Alexander K. Foote, Yuesong Hu, Jhordan Rogers, Khalid Salaita

**Affiliations:** Department of Chemistry, 1371 Emory University, 1515 Dickey Drive, Atlanta, Georgia 30322, United States

**Keywords:** T cell mechanobiology, DNA origami, tension sensor, single-molecule fluorescence, spectral multiplexing

## Abstract

A key event in triggering adaptive immunity is the binding of a T cell receptor (TCR) to its antigen at the T cell–target cell interface. Mechanical forces are critical for TCR–antigen interactions, where piconewton (pN) forces modulate immune responses. A major challenge in studying these interactions is quantifying forces at the single-molecule scale, as T cells can activate in response to just 1–10 antigen molecules. To address this, we developed single-molecule DNA origami tension sensors (smDOTS) for high-resolution force mapping. Our design includes spectral fingerprint density reporters, multiple quenchers for extended force dynamics monitoring, and tunable cholesterol anchors for controlled mobility. We report unprecedented measurements of TCR–antigen forces at fluid membranes, detecting forces with magnitudes of 8 to 19 pN, and tracking ligand translocation. Multiplexing enabled the simultaneous imaging of sensors with different force thresholds. This approach could further reveal bond lifetimes and force dynamics, deepening our understanding of TCR-mediated signaling.

## Graphical abstract

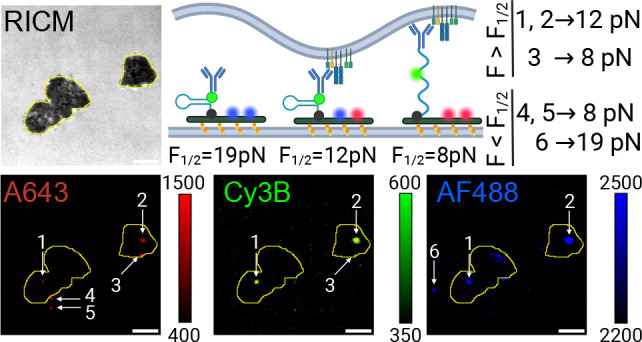

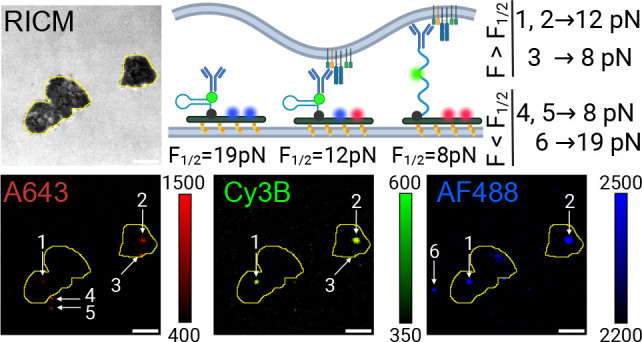

To fight cancer and viral infections, the T cell receptor (TCR) scans the surface of target cells, identifying abnormal peptides presented by major histocompatibility complex (pMHC) molecules.^1^ This recognition occurs through a *juxtacrine* interaction, a form of cell-to-cell communication requiring direct *physical* contact between the T cell and the target cell.^2^ Because TCR–antigen binding occurs at the junction of two dynamic cells, many have postulated that the complex experiences mechanical forces.^3–10^ Using molecular tension sensors,^11,12^ we showed that the TCR transmits 10–15 piconewtons (pN) of force upon antigen engagement and mechanical tension plays a crucial role in T cell activation and signal transduction.^9,13,14^ T cell coreceptors also engage in force transmission.^9,14–17^ Complementary single-molecule techniques such as AFM,^5^ biomembrane force probes,^13,16^ and optical tweezers^18^ showed mechanical sensitivity of the TCR. DNA-based molecular tension sensors^9,19,20^ are comprised of a DNA hairpin modified with the fluorophore–quencher pair anchored to a surface and presenting specific ligands. Under force, the hairpin unravels, separating the fluorophore from the quencher and generating a massive increase in the fluorescence emission. Recently, we reported a DNA origami tension sensor (DOTS) probe that can be tethered to lipid membranes to investigate TCR forces at fluid intermembrane junctions.^14^ DOTS are composed of a 40 × 80 nm rectangular sheet that presents a DNA tension probe on the top face while the bottom face presents single stranded DNA “legs” that allow the structure to embed into fluid membranes using cholesterol anchors. DOTS are also labeled by a second fluorophore that is used for density reporting. This reporter fluorophore must be spectrally separate from the force indicating fluorophore, spaced at distances exceeding the Förster radius to prevent unwanted FRET. At the immune synapse, TCRs engage antigen-loaded DOTS, applying forces that pull the DNA hairpins open. Unlike rigid substrates, DOTS move laterally within the membrane, enabling the measurement of forces in a dynamic, fluid context. This mobility mimics physiological conditions, allowing the study of transient TCR forces and clustering behavior in the immune synapse.

Given that T cells activate in response to as few as 1–10 antigens on the target cell surface, it is highly desirable to use single-molecule methods for the analysis of individual TCR–antigen forces.^21^ It is generally very difficult to capture the transient mechanical events generated by TCRs and hence previous studies used high probe density^14^ which is particularly problematic when studying rare and biologically significant events. For example, high-affinity TCR–pMHC interactions, which may play outsized roles in determining T cell activation outcomes, can be overshadowed in bulk measurements by more common, low-affinity TCR–pMHC interactions.^22^ Similarly, physical processes such as TCR clustering, force-induced conformational changes, and catch bonds become obscured in ensemble measurements.^23^

Single-molecule techniques overcome these challenges, allowing one to track individual TCR–pMHC interactions.^22,24,26^ To date, there have been a handful of studies using single-molecule methods to study the signal generated by molecular tension probes and these focused on forces applied by integrin receptors.^27–33^ Indeed, we and others used single-molecule methods to generate super-resolution maps of integrin forces.^28,34^ Past work focused on integrin forces because those events are long-lived and display large force magnitudes in comparison to TCR forces which are weak, infrequent, and transient. The only single-molecule investigation of TCR forces was reported by Schütz and colleagues, where they measured TCR–ligand forces at the single-molecule level using a FRET-based sensor.^23^ Surprisingly, the team concluded that there are no forces transmitted by the TCR when antigens are presented on a fluid membrane. This conclusion was confounding, as our recent work with DOTS showed TCR–pMHC forces exceeding 8 pN at cell–cell and cell–membrane junctions.^14^ Possible explanations for the discrepancy may be the use of analogue low sensitivity tension sensors, the low diffusion coefficient of DOTS, and the biological differences between CD4 and CD8 T cells. To reconcile this discrepancy, here, we reengineered DOTS for single-molecule analysis to take advantage of the enhanced sensitivity of DNA tension probes for the mechanical detection of TCR–antigen interactions.

The initial design of DOTS was optimized for bulk measurements; therefore, two main modifications were introduced to enable single molecule DOTS (smDOTS) imaging. The first pertained to the reporter fluorophores, which we refer to as spectral fingerprinting dyes (Figure 1A–B). We increased the number of these dyes to four to enhance signal allowing for longer duration tracking due to reduced photobleaching.^35^ We also introduced different types of dyes as barcodes to multiplex signal readout from unique structures. The second was modification of the tension reporter, and here we incorporated three quenchers to suppress the false tension signal caused by defects in hybridization or labeling, further protecting the force probe from photobleaching (Supplementary Note 2.1). The three quenchers were placed near the base of the hairpin at a 5 nm distance from one another. The force-indicating fluorophore was conjugated to the top strand, which binds to the upper part of the hairpin strand and presents a biological ligand (Figure 1A–B).

**Figure 1:**
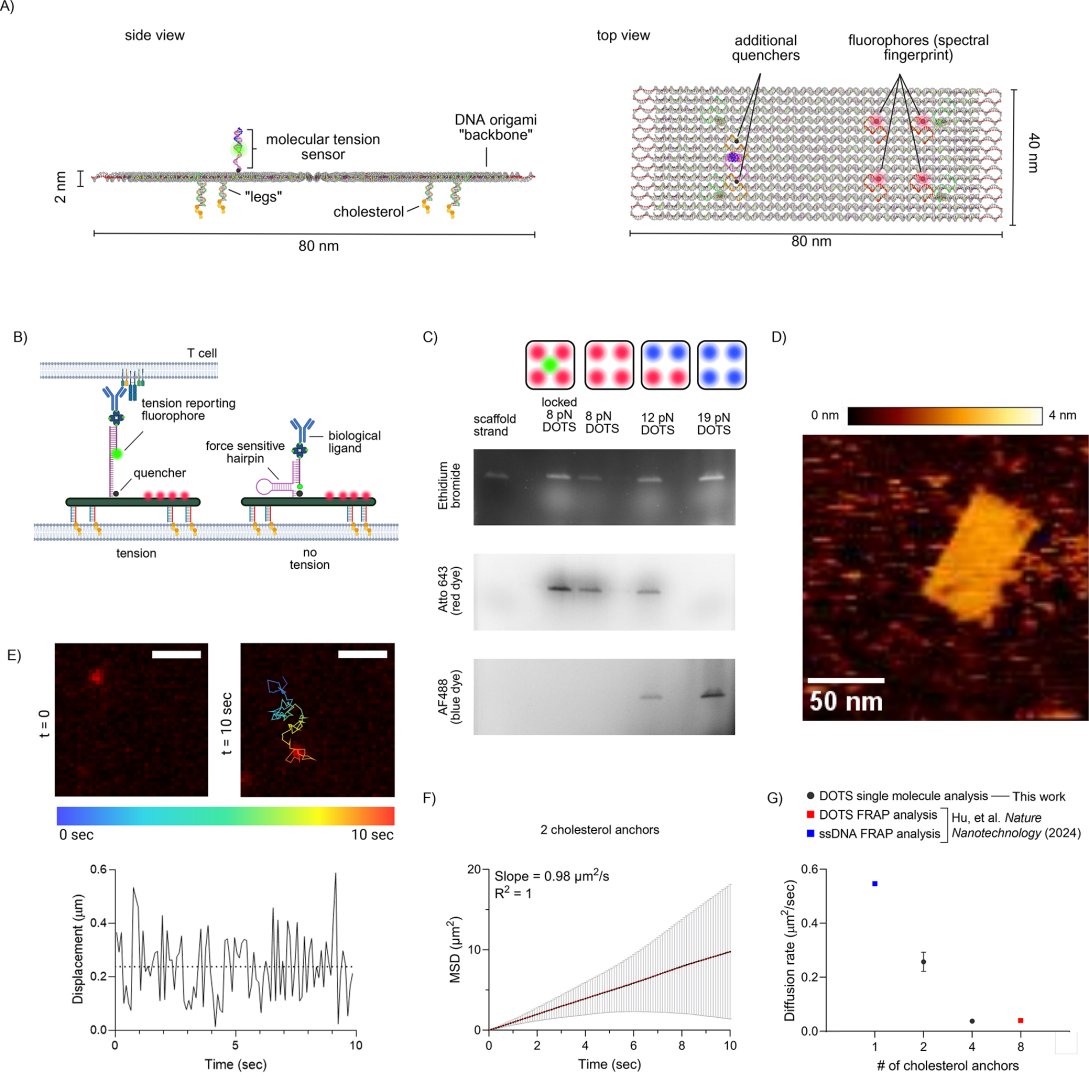
Overview and characterization of smDOTS. (A) Side and top Oxview schematic of smDOTS structure: a 40 × 80 nm DNA origami base, a tunable number of legs with complementary cholesterol anchors, four spectral fingerprinting dyes, and the molecular tension sensor. (B) Schematic further illustrating the mechanism of force sensing. The molecular tension sensor is made up of a DNA hairpin which unfolds under the external application of tension and is flanked by a fluorophore and quencher. The hairpin presents a biological ligand for interaction with TCR. (C) Characterization and purification via gel electrophoresis. From left to right, the lanes contain: the scaffold strand used as a control to confirm the relative size of the constructs, the 8 pN smDOTS construct annealed with a locking strand to simulate tension, the 8 pN smDOTS construct without the locking strand, 12 pN smDOTS, and 19 pN smDOTS. The schematic above each lane represents which fluorophores were used to tag the associated construct. Gels were imaged in three channels corresponding to the nucleic acid stain (ethidium bromide), the red dye (Atto643), and the blue dye (AF488). (D) Tapping mode AFM image of smDOTs on mica. (E) Representative total internal reflection fluorescence (TIRF) microscopy data showing the trajectory of a single particle with two cholesterol anchors. Scale bar = 2 μm. The colored line represents the location of the particle at a given time, ranging from 0 to 10 s. Displacement was plotted as a function of time, with the horizontal dotted line showing the median displacement. (F) Plot showing the mean squared displacement as a function of time. Data were collected from thousands of particle trajectories for the two cholesterol anchor construct. Error bars represent the margin of error from upper and lower bounds. (G) Plot representing the diffusion rate corresponding to a varying number of cholesterol anchors on DOTS. A higher number of cholesterol anchors results in slower particle diffusion. Data for two and four cholesterol anchors was obtained by tracking the trajectory of single particles as shown in (E) and (F), while the eight cholesterol anchor data and the single stranded DNA diffusion rates were determined using FRAP in our previous work.^14^ Error bars for single-molecule diffusion rates represent the standard error of the mean across three replicates.

Spectral fingerprinting allows us to identify and differentiate between multiple molecules based on their unique spectral characteristics. The reason for using four fluorophores for spectral fingerprinting is to increase the photon budget, as these dyes will be imaged constantly for particle tracking. Cy3B was used as the force indicating fluorophore and is only visible when *F* > *F*_1/2_. Atto 643 (A643) and AlexaFluor 488 (AF488) were used as spectral fingerprinting dyes to identify all particles, whether they experience force or not. As a proof of concept, we created three unique smDOTS (Figure 1C). The first presented a hairpin with a force threshold of 8 pN which was identified by a red spectral fingerprint (four Atto643 dyes). To contrast, we also created smDOTS with a 19 pN force probe identified by a blue spectral fingerprint (four Alexa488 dyes). Finally, the third structure presented a 12 pN force probe tagged using a blue-red fingerprint (two Alexa488 and two Atto643). While we focused on creating only three constructs, the potential exists to design and develop a greater variety of smDOTS. For example, further manipulation of the ratiometric signal of two or more dyes and incorporating FRET has enabled encoding dozens to several hundred species in single-molecule fluorescence techniques, such as fluorescence in situ hybridization (FISH), photoactivated localization microscopy (PALM), and DNA-points accumulation for imaging in nanoscale topography (DNA-PAINT).^36–39^

The design and assembly of smDOTS followed previous precedent in DNA origami assembly^14,40,41^ by combining the scaffold and staple strands (Supplementary Tables 3.1.1–3.1.3, Supplementary Figures 3.2.1–3.2.4) and annealing in Tris-acetate-EDTA (TAE) buffer. The structures were characterized and isolated using gel electrophoresis (Figure 1C, Supplementary Figures 3.2.5–3.2.7). Agarose gels showed an Atto643 signal on the 8 pN smDOTS, both Atto643 (A643) and Alexa Fluor 488 on the 12 pN smDOTS, and Alexa Fluor 488 (AF488) signal on the 19 pN smDOTS (Figure 1C). The constructs were then purified from the gel and further characterized via AFM to confirm the shape and dimensions of the nanostructure (Figure 1D, Supplementary Figure 3.2.8). An added benefit of the use of DOTS is the ability to tune the mobility of these nanostructures by tuning the number of cholesterol anchors. This is advantageous both for monitoring dynamic and fluid movements such as TCR clustering and for mimicking the physiological mobility of antigens. To characterize the mobility of these structures on fluid supported lipid membranes (SLBs), we created multiple constructs with varying numbers of cholesterol anchors (Figure 1E–G). We previously reported the diffusion rate of DOTS with 8 cholesterol anchors,^14^ while two and four cholesterol diffusion rates were measured by tracking the movement of single probes in an SLB. We also compared the diffusion of these constructs to that of single stranded DNA modified with cholesterol, which was reported in the literature.^14^ Time dependent single-particle tracking (Figure 1E) was repeated for thousands of particles to obtain the mean square displacement (MSD) as a function of time (Figure 1F, Supplementary MATLAB Script 4.1). We then computed the diffusion coefficient (*D*) using the following equation, where ν is the number of dimensions (in this case 2) and τ is the delay time.^42^ Here, α represents Brownian motion and is therefore equal to 1. Diffusion rates were measured from three independent replicates (Supplementary Figure 3.2.9).

1 $$MSD=2\nu D\tau^{\alpha }$$

This analysis indicated *D* = 0.26 ± 0.06 μm^2^/s and *D* = 0.04 ± 0.009 μm^2^/s for the two and four cholesterol anchors, respectively, and combined with previously reported *D* = 0.04 μm^2^/s for eight cholesterol anchors as well as *D* = 0.55 μm^2^/s for the single stranded cholesterol modified DNA to give Figure 1G. For the remainder of this study, we used DOTS with four cholesterol anchors, because these display a *D* within the range of physiological mobility (<0.1 μm^2^/s)^43–45^ and conveniently provide sufficient mobility for effective particle tracking, allowing high-precision single-particle analysis at our imaging frame rate (0.0167 Hz).

For spectral characterization, smDOTS constructs were loaded onto fluid SLBs and imaged by using total internal reflection fluorescence (TIRF) microscopy. Each sample was imaged in three fluorescent channels corresponding to the red dye (A643), the green dye (Cy3B), and the blue dye (AF488) (Supplementary Table 3.1.4). We used a TuCAM dual camera system (Supplementary Figure 3.2.10) to simultaneously image two channels. Briefly, a dichroic mirror was placed in the optical path to deflect light of wavelength <640 nm, comprising the emission of Cy3B and AF488, to the second camera. Following image registration, particles in each channel were identified using ThunderSTORM analysis,^46^ then sorted based on which channels they exist in using a custom Matlab algorithm (Supplementary Figure 3.2.11). Briefly, localizations from each channel were spatially compared with those from other channels, and any localizations within 500 nm (Supplementary Figure 3.2.12) were classified as belonging to the same particle (Supplementary MATLAB Script 4.2). We observed minimal bleedthrough or cross-excitation between different fluorescence channels, which was subsequently eliminated using intensity thresholding (Supplementary Figure 3.2.13). Particles which were present only in the red channel were identified as 8 pN smDOTS and particles present only in the blue channel were labeled as 19 pN smDOTS, while particles present in both the red and blue channels were determined to be 12 pN smDOTS (Figure 2A). Automated analysis of five replicate experiments localizing hundreds of particles on different surfaces correctly identified 98% of 8 pN smDOTS and 90% of 19 pN smDOTS particles; however, only 51% of 12 pN smDOTS were identified correctly (Figure 2B). The most likely cause for this reduction in fidelity relates to the movement of particles in the time frame needed to switch channels, which was required to capture emission from red and blue channels, as well as potential structural defects, leading to some particles being labeled by only red or only blue dyes. To verify our ability to identify individual constructs in a complex heterogeneous environment, we combined all three smDOTS constructs in a 1:1:1 stoichiometry and incubated them in a single sample. Here, we successfully identified all three types of smDOTS with population abundances of 61% A643, 24% AF488, and 7% of both A643 and AF488. That deviated from the 1:1:1 expected ratio and is consistent with lower fidelity identification of the 12pN smDOTS compared to the 8 and 19 pN counterparts (Figure 2A–B). Note that the number of particles identified in the red channel exceeds the number of particles in blue or green channels likely due to a combination of factors, including the limited photostability of AF488, the single fluorophore encoding in the green channel, and higher signal-to-noise ratio in the red channel.

**Figure 2:**
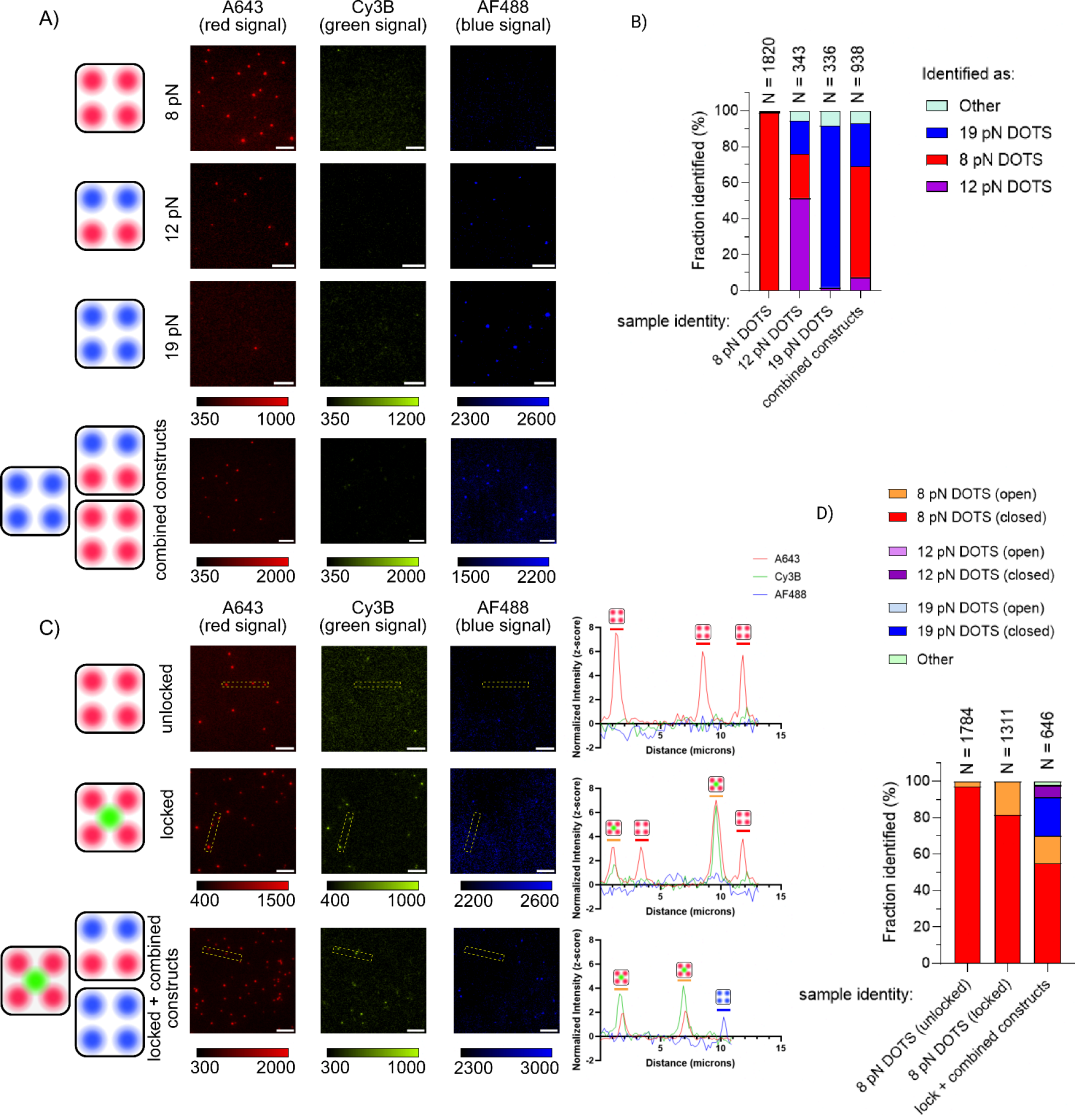
Spectral characterization of unique smDOTS constructs. (A) 8, 12, and 19 pN smDOTS imaged in three fluorescent channels. A fourth sample combining all three constructs was also imaged. (B) The ratios of particles identified in each sample type. Each column is an individual sample, with the identity noted below. The colors red, purple, and blue are used to indicate the fraction of particles identified by our algorithm as 8, 12, or 19 pN DOTS, respectively. In these plots, “other” represents signal in the green channel that is not colocalized with a spectral fingerprint. The number of particles in each population is indicated above. This plot represents pooled data from five replicates. (C) Representative images for locking strand experiment, used to simulate tension. The top row shows the 8 pN smDOTS construct without the locking strand. The second row shows the 8 pN construct with the locking strand. The sample shown in the third row contains the 8 pN construct with the locking strand, as well as 12 and 19 pN constructs with no locking strand. Yellow boxes indicate areas chosen for linescan analysis, shown to the right. (D) Fractions of particles identified in each sample. Each column is an individual sample, with the identity noted below. The number of particles in each population is indicated above. This plot represents pooled data from three replicates. For the unlocked 8 pN construct, 97% of red particles have no green colocalizations, meaning that the tension sensor is folded. The locked 8 pN construct shows that number of red-green colocalizations increased to 19%, indicating that these hairpins have been unfolded by the complementary locking sequence. Finally in the sample containing a mixture of constructs, red-green colocalizations are present, but there are no blue-green or red-blue-green colocalizations. This demonstrates our capability to distinguish between folded and unfolded tension sensors in addition to identification of particle type via spectral fingerprinting.

Next, we simulated force-induced hairpin opening events using a “locking strand” complementary to the stem–loop region of the 8 pN construct. The “locked” construct was annealed with the locking strand, which prevents the hairpin from folding and leads to separation of the fluorophore and quenchers, generating a turn-on signal in the green channel (Figure 2C). For samples annealed without the locking strand, only 3% of red particles had a corresponding green signal. When annealed with the locking strand, the red and green colocalizations increased to 19%, confirming binding of the locking strand to a subset of probes. Note that incomplete lock binding prevents the unfolding of 100% of hairpins. Next, we combined the locked 8 pN construct with the unlocked 12 and 19 pN constructs. This experiment emulates conditions where the forces applied exceed 8 pN, but are too weak to unfold the 12 or 19 pN hairpins. Here, we expect to see red and green colocalizations, but not blue and green or red, blue, and green. Indeed, all the green signal we observed which colocalized to a spectral fingerprint belonged to the 8 pN construct (15%), and we recorded no overlaps in green and blue or all three dyes (<1% combined) (Figure 2C–D). Thus, we showed that we could identify each construct and assign the observed tension signal with its corresponding hairpin threshold.

Next, we utilized smDOTS to measure and track forces generated by T cells in real time. Synthesized particles were embedded into an SLB and tagged with a biological ligand: either a pMHC loaded with a cognate SIINFEKL peptide or anti-CD3e antibody which binds to the CD3e subunit of the TCR complex. These surfaces were then seeded with transgenic CD8+ T cells that express a monoclonal population of TCRs that respond to SIINFEKL peptide antigen derived from ovalbumin (OVA). We started with 8 pN smDOTS which present pMHC (Figure 3A). In the red channel (A643) we observed smDOTS scattered throughout the SLB both within the cell and outside of it. Particles that were colocalized with a green signal (Cy3B) satisfied a set of stringent criteria (Supplementary Note 2.2) and were identified as smDOTS experiencing tension (Figure 3B). In Figure 3B–C we highlight a particle near the edge of the cell at three different time points. First, we noted signal only in the red channel, meaning that this was an 8 pN smDOTS particle experiencing <8 pN of force. One minute later, the red signal persisted, and a signal also appeared in the green channel. This showed that a TCR had engaged with the particle and exerted a force >8 pN. The green signal was transient and disappeared in the subsequent frame 1 min later, which indicates force termination. Thus, we have successfully detected forces generated by T cells at a single-molecule scale using a digital tension sensor. Interestingly, many cells exhibited a large highly fluorescent structure near the center of the cell. This is likely an aggregate of smDOTS due to TCR clustering and centralization^15,47^ and is consistent with our past observation^48^ of centralization of fluid antigens.^13,14^ To further examine this phenomenon, we used an anti-CD3 ligand which has a longer bond lifetime after binding to a TCR and imaged over the course of 10 min (Figure 3D–E). Here, we observed an aggregate of smDOTS near the center of the cell, as well as a single smDOTS particle near the edge of the cell. At the 3 min time point, this single particle engaged the cell, and we saw a turn-on signal in the green channel, indicating *F* > 8 pN. Following the initiation of force, the particle began to move closer to the aggregate at the center of the cell (Figure 3E). We measured the instantaneous speed of this particle at each time point. While the initial speed of the particle was <500 nm/min, following force initiation the speed increased to 100–1500 nm/min for a duration of up to 3 min, before slowing back to initial speed as the particle neared the mass at the cell center (Figure 3F). These results are consistent with reports of TCR transport rates.^49^ The initial speed measured here is slower than the diffusion rate demonstrated in Figure 1G. The reduction in speed is likely an indicator of particle binding.^22^ In addition to the 8 pN smDOTS, we also tested 12 and 19 pN smDOTS which presented pMHCs to interact with TCRs. In both cases, we detected the occurrence of force events (Figure 3G–H), though we found that force events exceeding 19 pN were rare and difficult to measure. Additional examples of force measurements (Supplementary Figure 3.2.14) and ligand translocation following force events (Supplementary Figure 3.2.15) are provided in the Supporting Information document. To test the multiplexing capability of our design, we included all three smDOTS particles on one surface to detect forces of different magnitudes simultaneously (Figure 3I). Each particle was identified based on the spectral fingerprints (Figure 3J). Force events were identified based on the green (Cy3B) signals of particles. The multiplexing capabilities demonstrated here are limited, particularly due to the low identification rates of the 12 pN construct. To improve the identification of structures presenting more than one organic dye, we suggest that future studies increase the number of fluorophores in each construct. Nonetheless, we have demonstrated that our approach does support multiplexing in at least two channels, with potential for more, despite the need for further optimization. This approach opens numerous possibilities for understanding the role of mechanical forces generated by T cells. For example, future studies may focus on localizing the magnitude of forces with known mechanotransducing motifs or cell functions. Alternatively, cells may be treated with external stimuli to investigate how they impact the magnitude or duration of forces. Here, we used spectroscopic multiplexing for the simultaneous measurement of force events with various magnitudes.

**Figure 3:**
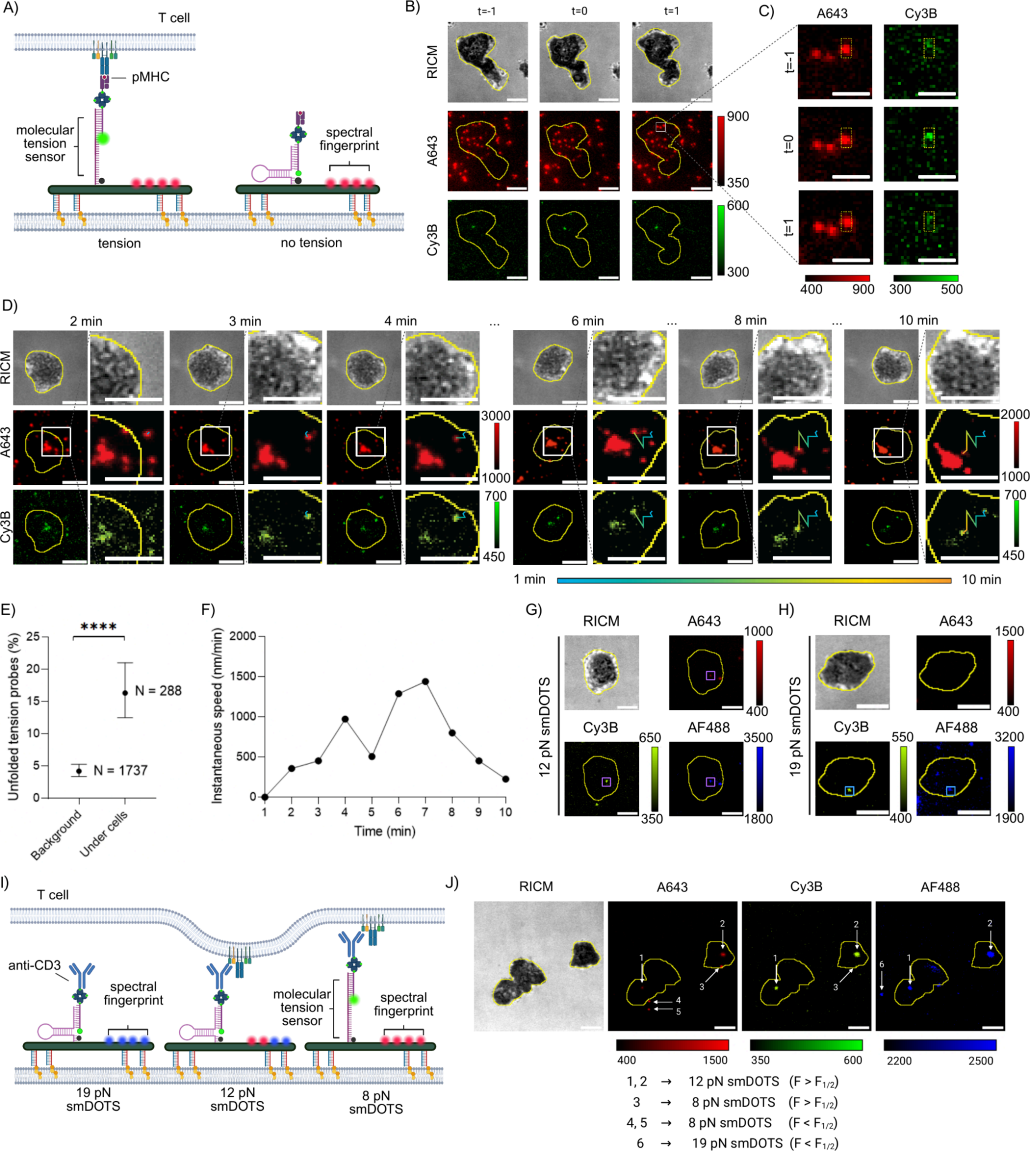
smDOTS measurement and tracking mechanical forces transmitted by TCRs. (A) Schematic illustrating experimental setup for (B) and (C). Eight pN smDOTS presenting a pMHC are loaded onto a SLB. TCRs engage the pMHC and generate tension forces, resulting in hairpin unfolding and a turn-on Cy3B signal. (B) Example of smDOTS interacting with cell and real-time tension measurements. (C) Inset of (B) showing particle of interest. A643 signal persists across all time points. Cy3B signal is present and colocalized during the second frame only, indicating transient forces. Scale bar = 2 μm. (D) Time-lapse images showing T cells interacting with smDOTS over a 10 min period. The white box in each image indicates the region shown in the corresponding zoomed-in panel to the right. The trajectory of a single particle near the edge of the cell is tracked and color-coded to indicate time, with the time bar spanning from 1 to 10 min. At the 3 min time point, the particle experiences a force greater than 8 pN and is subsequently translocated toward the center of the cell. (E) Fraction of particles which demonstrate colocalization of the spectral fingerprinting dye (A643) and the force indicating fluorophore (Cy3B). In the background, <5% of particles are colocalized, meaning that there is minimal spontaneous unfolding of the hairpin. Under cells, the fraction of colocalized particles increases, indicating that interactions with cells lead to hairpin unfolding. Measurements of *N* = 1737 and *N* = 288 particles were collected in the background and under cells, respectively, from three biological replicates. Error bars indicate the upper and lower limits of the 95% confidence interval. (F) Plot showing instantaneous speed of the particle of interest from (D) at each time point. The particle moves faster following force initiation before slowing as it nears the center of the cell. (G) Representative images demonstrating tension when T cells interact with 12 pN smDOTS presenting pMHC. (H) Representative images demonstrating tension when T cells interact with 12 pN smDOTS presenting pMHC. (I) Schematic illustrating experimental setup in (J). All three smDOTS constructs are loaded onto a single SLB and present pMHC for interaction with T cells. (J) Representative images showing surfaces previously described. Each type of construct is identified by its spectral fingerprint, allowing for the quantification of force magnitudes for individual TCR–pMHC interactions. All scale bars shown represent 5 μm unless otherwise stated.

In this study, we optimized DOTS for single-molecule studies, tuned the mobility, and introduced spectral fingerprinting. We then used smDOTS to measure TCR–antigen forces on the single-molecule scale. Contrary to conclusions made by Schütz and colleagues,^23^ we detected forces ranging from 8 to 19 pN at fluid interfaces. This discrepancy is likely due to the enhanced sensitivity of our digital tension probe. In addition, we observed translocation of antigens following TCR engagement, as is consistent with findings by Groves et al.^22,49^ This work demonstrates the potential for using smDOTS to answer unique questions regarding biological activity in complex environments. For example, combining this approach with the other probes we have developed may yield information about the force loading rate.^27^ In addition, spectral fingerprinting can be used to identify smDOTS constructs that present different biological ligands. This approach allows us to observe and quantify preferential interactions between cells and specific ligands, providing valuable insights into cellular behavior, receptor–ligand specificity, and signal transduction pathways. Such studies can be particularly useful in understanding cellular responses to different extracellular cues, probing mechanisms of selective adhesion, and identifying key ligands involved in processes such as immune recognition, cancer metastasis, and tissue regeneration. By expanding the use of smDOTS in this manner, we can develop a powerful tool for studying molecular interactions at the single-molecule level across a wide range of biological systems.

## Supplementary Material



## Notes

Additional experimental details, materials, and methods; full DNA sequences; analysis algorithms; supplementary figures and additional references; and timelapse videos (PDF)

## Abbreviations

TCR: T cell receptor
DOTS: DNA origami tension sensor
SLB: supported lipid bilayer
pMHC: peptide-major histocompatibility complex
pN: piconewton
smDOTS: single-molecule DNA origami tension sensor
FRET: Förster resonance energy transfer
TIRF: total internal reflection fluorescence
OVA: ovalbumin
A643: Atto 643
AF488: AlexaFluor 488
MSD: mean square displacement
FISH: fluorescence in situ hybridization
PALM: photoactivated localization microscopy
DNA-PAINT: DNA-points accumulation for imaging in nanoscale topography
