# Supplementary material for: DNA Origami Tension Sensors (DOTS) for Single-Molecule Force Measurements at Fluid Intermembrane Junctions
Source: Nano Lett. 2025 Aug 25;25(36):13419–27. doi: 10.1021/acs.nanolett.5c02130 (PMC12426984; doi:10.1021/acs.nanolett.5c02130)
Supplement: Supplementary file 1 [file nl5c02130_si_001.pdf]

Supporting Information

DNA Origami Tension Sensors (DOTS) for Single Molecule Force Measurements at Fluid Intermembrane Junctions

*Sarah Al Abdullatif, Alexander K. Foote, Yuesong Hu, Jhordan Rogers, Khalid Salaita**

*Corresponding author: k.salaita@emory.edu

Table of Contents

1. Materials and Methods……………………………………………………………..…...……1

1.1. Reagents……………………..……………………………………...…..………1

1.2. T cell sourcing…………………………………………………...…...……..2

1.3. Instrumentation……………………………………………………………….…2

1.4. Oligonucleotide modification………………………………….………………..3

1.5. Production of DNA origami scaffold……………………………………………4

1.6. Origami synthesis and purification……………………….…………………..…….5

1.7. SLB preparation…………………………………………..………………..6

1.8. Data Analysis……………………………..……………………….……….……7

2. Supplementary Notes……………………………………………………………………...8

2.1. Determining the efficiency of additional quenchers..8

2.2. Criteria for correct identification of force measurements…………………………...14

3. Supplementary Tables and Figures ……………………….…………….………....………15

3.1. Supplementary Tables……………………………………..…….…………..15

Table 3.1.1. Modified DNA sequences used for smDOTS………………………..15

Table 3.1.2. Masses of staple strands labeled by spectral fingerprint dye ………...17

Table 3.1.3. List of 77 unmodified DNA staple sequences………..……………...18

Table 3.1.4. Optical configuration for imaging channels……………………........20

3.2. Supplementary Figures………………...………………..……………..……………..21

Figure 3.2.1. HPLC chromatograms for BHQ modified staple strands and Cy3B labeled top strand……………………………………………………………....21

Figure 3.2.2. HPLC chromatograms for Atto 643 labeled staple strands………....22

Figure 3.2.3. HPLC chromatograms for AlexaFluor 488 labeled staple strands.....23

Figure 3.2.4. Agarose gel characterization of origami scaffold strand…………....24

Figure 3.2.5. Full gel images representing ethidium bromide staining of nucleic acids...25

Figure 3.2.6. Full gel images representing Atto 643 dye labeling………………...26

Figure 3.2.7. Full gel images representing AF488 dye labeling………………......27

Figure 3.2.8. AFM images of multiple smDOTS structures………………………28

Figure 3.2.9. Diffusion rates of smDOTS measured from three independent replicates……………………………………………………………………....29

Figure 3.2.10. Optical configuration for fluorescence imaging………………....30

Figure 3.2.11. Flowchart of data analysis workflow………………..……………31

Figure 3.2.12. Determination of nearest-neighbor distances for identification of colocalized particles…………………………………………………………...32

Figure 3.2.13. Quantification of bleedthrough and noise across three fluorescence channels……………………………………………………….…………33

Figure 3.2.14. Additional examples of tension measurements……………………35

Figure 3.2.15. Additional examples of ligand translocation following force activation………………………………………………………………36

4. MATLAB scripts……………………………………………...……………………37

4.1. Particle tracking algorithm…………………………………………...……...37

4.2. Sorting algorithm……………………………………..…………………...43

5. References……………………………………………………………………………..57

1. Materials and Methods

1.1. Reagents

DOPC (catalogue number 850375C, 200 mg), Ni-NTA-DGS (catalogue number 790404C, 5 mg), DPPC (catalogue number 850355C, 25 mg) and 18:1 Biotinyl Cap PE (catalogue number 870273C, 25 mg) were purchased from Avanti Polar Lipids. Tris (1 M, catalogue number AM9856), EDTA (0.5 M, catalogue number AM9260G), $MgCl_2$ (1 M, catalogue number AM9530G) were purchased from ThermoFisher. Bovine serum albumin (BSA) (catalogue number 10735078001), 100 kDa Amicon ultra-0.5 centrifugal filter (catalogue number UFC510096), 2x YT (catalogue number Y2377), and Hank's balanced salt solution (H8264-6X500ML) were purchased from Sigma Aldrich. Cy3B-NHS ester and AF488-azide were purchased from Lumiprobe (Hunt Valley, Maryland). Atto643-NHS ester was purchased from Atto-Tec. Milli-Q water was obtained from a Nanopure system with 18.2 M-1 -cm resistivity. All oligonucleotides were purchased from Integrated DNA Technologies (Coralville, IA) and were purified either by reverse phase HPLC or standard desalting. Agarose (catalog number R0491) was purchased from Thermo Scientific. Streptavidin (S000-01) was purchased from Rockland-Inc (Pottstown, PA). ProPlate® Microtiter (204969) are purchased from Thermo-Fisher Scientific. Biotinylated pMHC ovalbumin (SIINFEKL) was obtained from the NIH Tetramer Core Facility at Emory University. P2 size exclusion gel (catalogue number 1504118) was purchased from Bio-Rad. Syringes (3 ml) were purchased from BD Biosciences. Cell strainers (catalogue number 15-1100) were bought from Biologix. Midi MACS (LS) start-up kit (separator, columns, stand, catalogue number 130-042-301) and mouse $CD8^+$ T cell isolation kit (catalogue number 130-104-075) were purchased from Miltenyi Biotec. Biotinylated anti-CD3 was purchased from Biolegend (San Diego, CA). Streptavidin (Cat# S000-01) was purchased from Rockland Immunochemicals Inc. (Rockland,

NY). His-ICAM-1 (catalogue number 50440-M03H) was purchased from Sino Biological. All other reagents and materials (unless otherwise stated) were purchased from Sigma-Aldrich and used without purification. All buffers were prepared with 18.2 MΩ nanopure water. Tetracycline, XL1-Blue and p7560 phage were provided by Dr. Yonggang Ke and Dr. Luyao Shen.

1.2. T cell sourcing

The OT-1 transgenic mice are housed at the Division of Animal Resources Facility at Emory University. All the experiments were approved and performed under the Institutional Animal Care and Use Committee (IACUC) protocol. Protocol number: PROTO201800239. Naive OT-1 T cells were isolated from the spleen using magnetic activated cell sorting, as instructed by the manufacturer's $CD8^+$ T cell Isolation Kit (Miltenyi Biotec). In brief, a single cell suspension of splenocytes was obtained and incubated with biotinylated antibodies targeting unwanted splenic cell populations. These populations were separated from the OT-1 T cells using anti-biotin magnetic beads and enrichment on a magnetic column. The purified T cells were then washed, suspended in Hank's balanced salt solution (HBSS) solution and kept on ice until the experiment.

1.3. Instrumentation

The microscope used was NSTORM Nikon microscope (Ti E motorized inverted microscope body), operated by Nikon Elements software. A CFI Apo 100X NA 1.49 objective was used. The optical system includes a total internal reflectance fluorescence (TIRF) variable mirror launcher and a Nikon Perfect Focus System, an interferometry- based device which corrects z-drift of the stage. Reflection interference contrast microscopy (RICM) images were captured using a filter cube (Chroma 97270 SRIC C168785) and by a Lumen Dynamics X-Cite 120 LED light source. A Chroma Quad (Chroma TRF89901 ZT405/488/561/640rpc) TIRF filter cube was used in the

imaging acquisition of all three fluorescence channels. The optical configuration included the Andor TuCAM dual camera system comprised of two Andor DU-897 X-9319 cameras. A Chroma ZT647rdc dichroic mirror was placed in the optical path to deflect light of wavelength < 640 nm, comprising the emission of Cy3B and AF488, to the second camera. All ultrapure water was obtained from a Barnstead Nanopure water purifying system (Thermo Fisher) that indicated a resistivity of 18.2 MΩ. Nucleic acid purification was performed using a high-performance liquid chromatography (HPLC, Agilent 1100) equipped with a diode array detector. Microvolume absorbance measurements were obtained using a Nanodrop 2000 UV-Vis Spectrophotometer (Thermo Scientific). Mass spectrometry was conducting using a Thermo scientific LTQ Velos Orbitrap MS. DNA constructs were annealed using T100 thermal cyclers (Bio-Rad). Gel electrophoresis was conducted using a Mini-Sub cell GT and PowerPac Basic (Bio-Rad) and visualized using an iBright 1500 (Thermo Scientific).

1.4. Oligonucleotide modification

The modification of DNA strands has been adapted from our previously published work[1], but is described here for the benefit of the readership.

Oligonucleotide–dye conjugates were prepared by coupling the amine on the DNA strand with the activated NHS ester of the organic dye. In the case of AF488 labelled oligonucleotides, the amine on the DNA strand was first reacted with DBCO-NHS ester, followed by a strain-promoted click reaction with AF488-azide. Briefly, aminated DNA strands (100 µM) were mixed with excess BHQ2-NHS ester, Cy3B-NHS ester, Atto643 NHS ester or DBCO NHS ester (500 $\mu g\ ml^{-1}$) and allowed to react in aqueous solution (pH 9) for 1 h at room temperature. The mixture was then filtered through P2 gel to remove salts and unreacted organic material and then purified by HPLC with an Agilent AdvanceBio Oligonucleotide C18 column (4.6 × 150 mm, 2.7 µm) (**Figures 3.2.1.**

and 3.2.2). Mobile phases A (0.1 M triethlyamine acetate) and B (acetonitrile) were used for a linear gradient elution of 10–100% B over 50 min at a flow rate of 0.5 ml min^{-1}. Oligonucleotides conjugated to DBCO were immediately isolated then resuspended in 1x PBS and added to the azide modified AF488 (**Figure 3.2.3**). This reaction proceeded for 3 hours followed by a second round of HPLC purification. The desired products were characterized by electrospray ionization mass spectrometry (**Table 3.1.2**).

1.5. Production of DNA origami scaffold

The single stranded DNA p7560 scaffold was prepared using a standard protocol[2], which is described here for the reader's convenience.

Day 1: Preparation of media and overnight cell culture

We prepared 2x Yeast Extract Tryptone (YT) microbial growth medium by dissolving 24.8 g of 2x YT in 800 mL of water. Luria Broth (LB) media was prepared by dissolving 1.75 g of LB in 70 mL of water. In addition, we produced 50 mL of 1 M $MgCl_2$ and 50 mL of 10 mM Tris base. All solutions were autoclaved prior to use. For preparation of the bacterial culture, we added 2 mL of LB media to a bacterial growth tube, followed by 20 uL of XL1-Blue and tetracycline (10 μg/mL). The bacteria was incubated at 37 °C while shaking at 250 rpm overnight.

Day 2: Amplification and harvesting of phage DNA

2 mL of the bacterial culture was added to 200 mL of 2x YT media, then incubated at 37 °C while shaking at 250 rpm for 2 hours. After two hours, we measured the absorbance at 600 nm. This was done at 30 min intervals until the OD_{600} reached 0.5 (approximately $4x10^8$ cells/mL). Then, we added 50 uL of p7560 phage (stock concentration = $1x10^9$ pfu/μL) and 1 mL of 1M $MgCl_2$. This bacterial culture was then returned to 37 °C while shaking at 250 rpm for 2 hours, after which the

culture was transferred to 50 mL tubes and centrifuged at 8000 RCF for 20 min at 4 °C. This separated the bacteria, which precipitated, from the phage which remained in solution. To the supernatant, we added NaCl (3% w/v), PEG 8000 (4% w/v), then stored overnight at 4 °C.

Day 3: Isolating and collecting scaffold DNA strand

The solution containing phage was centrifuged at 10,000 RCF for 20 min at 4 °C. The supernatant was discarded, while precipitated phage was resuspended in 2 mL of 10 mM Tris, then centrifuged at 10,000 RCF for 10 min at 4 °C. Centrifugation was repeated 3 times, transferring the supernatant to a new tube each time. Then, we added 2x the remaining volume of PPB2 lysis buffer (0.2 M NaOH, 1% SDS), mixed, and incubated for 2 min. We neutralized the solution with 1.5x volume of PPB3 (3 M KOAc titrated to pH 5.5 with glacial acetic acid), then incubated on ice for 20 min. This was then centrifuged at 10,000 RCF for 15 min at 4 °C. The supernatant was collected and filtered over a 0.45 μm filter. Then, we added 1 volume of 100% ethanol and stored at -80 °C for 15 minutes, after which the solution was transferred to new tubes and centrifuged at 10,000 RCF for 15 minutes at 4 °C. We discarded the supernatant, washed the remaining precipitate with 10 mL of cold 75% ethanol, then centrifuged again. We discarded the supernatant and collected the precipitated DNA, which was resuspended in water. Finally, we characterized the product using agarose gel (**Figure 3.2.4**).

1.6. Origami synthesis and purification

The preparation of origami constructs has been adapted from our previously published work[3], but is described here for the benefit of the readership.

smDOTS constructs were assembled by mixing p7560 DNA scaffold strand (10 nM), four anchor strands (100 nM), BHQ-modified DNA hairpin strand and two additional BHQ modified strands

(600 nM), dye-modified density reporter strand (600 nM), dye-modified ligand strand (1,500 nM) and other 77 staple strands (300 nM) in folding buffer (5 mM Tris, 1 mM EDTA, 8 mM $MgCl_2$). This mixture was annealed by heating the mixture at 90 °C for 15 min and cooling down to 4 °C at a rate of 1 °C min^{-1}. Afterwards, the structures were purified by agarose gel electrophoresis (0.75% agarose gel, 0.5× TBE buffer, 8 mM $MgCl_2$). Agarose gel was run on ice for 2 h at 70 V and stained with ethidium bromide.

1.7. SLB preparation

The preparation of SUVs and SLBs has been adapted from our previously published work[3,4], but is described here for the benefit of the readership.

Lipids were combined in a round-bottom flask with a ratio of 98% DOPC, 2% DGS NTA. The lipid mixture was dried using a rotary evaporator to remove the chloroform. The lipids then hydrated with PBS to a concentration of 2 mg ml^{-1}. Three cycles of freeze–thaw were performed to disrupt large, multilamellar vesicle suspensions. The resulting small unilamellar vesicles (SUVs) were then repeatedly extruded through an 80 nm polycarbonate membrane filter at least ten times and stored at 4 °C before use.

The wells of optically transparent glass-bottom 96-well plates (ThermoFisher) were washed with 5 ml ethanol and water and etched with 6.5 M NaOH for 1 h at room temperature. The etched wells were washed with 10 ml water and treated with 100 µl 0.5 mg ml^{-1} SUVs for 5 min. After treatment, unbounded vesicles were removed by washing with 5 ml PBS. SLBs were subsequently blocked with bovine serum albumin (BSA, 0.05% in PBS) for 30 min and washed with 5 ml PBS. Then, cholesterol DNA strands (250 nM) were added to the SLB, incubated for 1 h and rinsed with PBS. Subsequently, DOTS (3 pM) were added for 1 h to bind to cholesterol strands on the SLB.

The wells were then washed with PBS supplemented with 8 mM $MgCl_2$ to remove excess DOTS. Streptavidin (5 μg ml^{-1}) was added to the SLB, incubated for 45 min and then washed with PBS supplemented with 8 mM $MgCl_2$. Subsequently, pMHC ligand (5 μg ml^{-1}) or biotinylated anti-CD3 antibody was added to the SLB, incubated for 45 min and washed using PBS supplemented with 8 mM $MgCl_2$. Finally, His-tagged ICAM-1 (1 μg ml^{-1}) was added for 1 h (resulting in a molecular density of ~60 molecules per μm^2). Wells were then washed with PBS supplemented with 8 mM $MgCl_2$ before adding cells.

1.8. Data Analysis

Image analysis was performed using Fiji software. Initially, image registration was conducted using the Fiji plugin Fast4DReg (F4DR). The Legacy 2D function F4DR Estimate Drift function was used. Particles that overlapped in more than one channel were identified manually, then selected in a ROI to be used as a reference. Time averaging was disabled, and default options were used for all other settings. Particle localization was performed using the Fiji plugin Thunderstorm[5] in each fluorescence channel. For this analysis, the camera settings were input to convert values to nm and photons. The pixel size is 160 nm for all three fluorescence channels. For the red channel, photoelectrons per A/D count is 16.5 while the base level of A/D counts is 179. This channel has an EM gain of 200. Meanwhile, for the green and blue channels, the photoelectrons per A/D count is 15.1 and the base level is 166. The green channel has an EM gain of 200 and the blue channel has an EM gain of 300. The analysis uses a wavelet filter (B-spline) to identify peaks. To approximate the localization of molecules the method used was detection of local maxima, with a peak intensity threshold of std(Wave.F1)*2 for red and green channels, or std(Wave.F1)*1.3 for the blue channel. For all other settings we used the default Thunderstorm conditions. The output

of this analysis underwent sorting via a MATLAB algorithm to categorize particles into their respective spectral identities. This workflow is detailed in **Figure 3.2.11**.

2. Supplementary Notes

2.1. Determining the efficiency of additional quenchers

OxDNA modeling approximates the distance between the fluorophore and each additional quencher averaging at 5.016 nm.

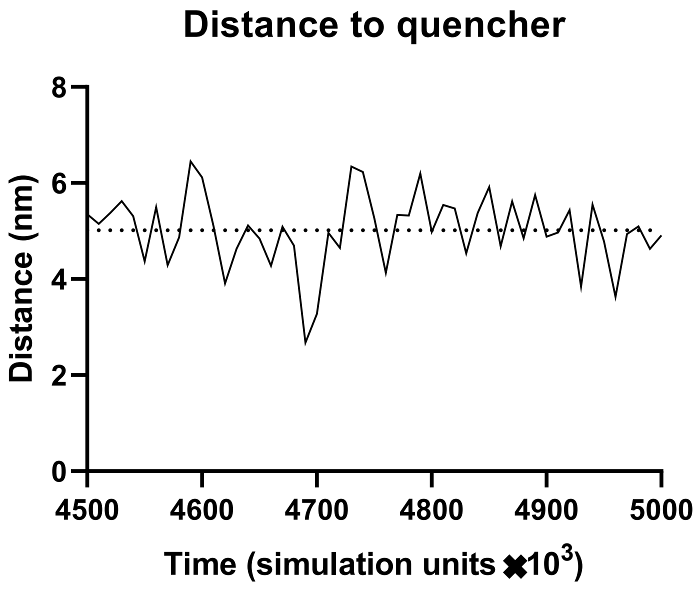

Shown below is the relaxed structure, with the positions of additional quenchers marked by a black circle and the position of the fluorophore (Cy3B) indicated by a glowing green circle. Distances between fluorophore and quencher, indicated by grey lines, were measured in simulation units and converted to nanometers.

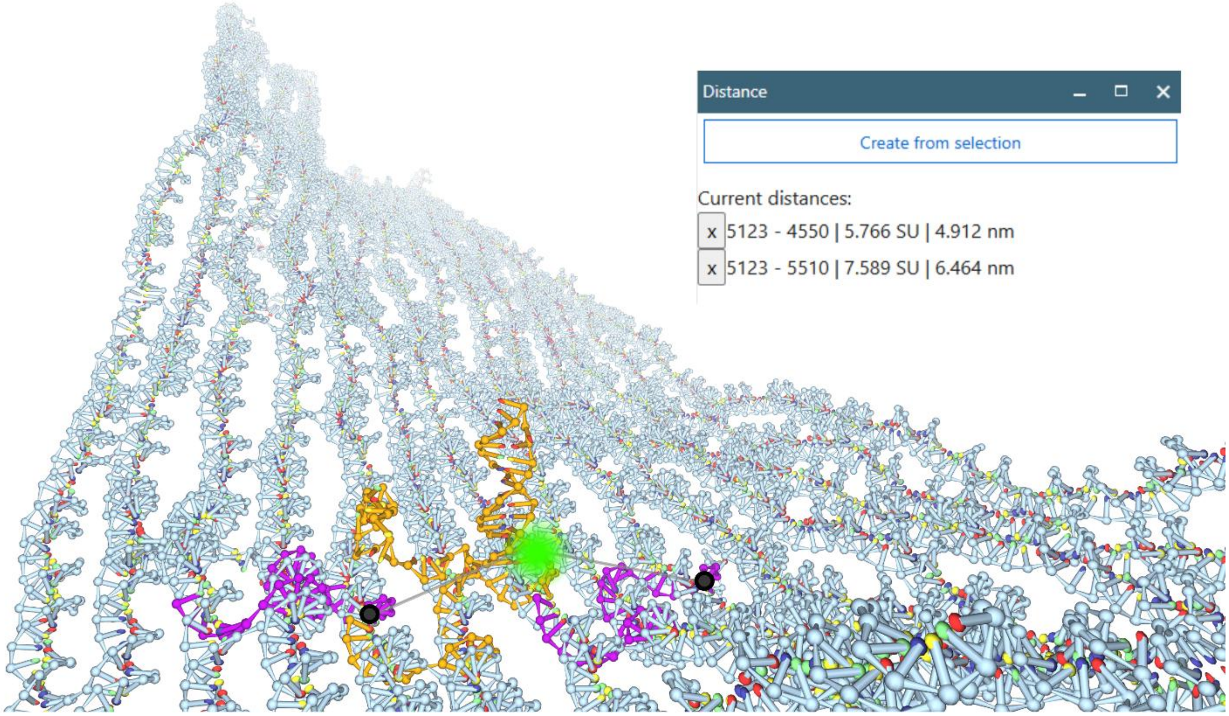

The FRET pair Cy3B and BHQ-2 have a Forster radius of 60.78 Å[6]. A single acceptor at 5 nm distance would result in 75.99% energy transfer efficiency. Furthermore, literature suggests that having multiple accepters for a FRET donor results in increased distance sensitivity[7–10]. Fabian, et al.[10] approximate the effects of multiple acceptors on energy transfer efficiency through the following equation, where n represents the number of acceptors, E_0 represents the original transfer efficiency for a single acceptor, and E_n represents the efficiency after an n-fold increase in the rate of transfer.

$$E_n = \frac{nE_0}{1 + (n-1)E_0}$$

Following this rationale, two quenchers at the approximated distances result in a FRET efficiency of 86.36% even if the central quencher fails.

2.2. Criteria for correct identification of force measurements

Our identification of tension events is supported by stringent criteria designed to maximize specificity and minimize false positives. Specifically, force events (green Cy3B signal) were only classified as true positives if they:

I. **Colocalized with at least one spectral fingerprinting dye (red and/or blue)** - to confirm probe identity and rule out nonspecific fluorescence. Signal in the green channel is only considered tension if it colocalizes with a spectral fingerprinting dye (red and/or blue). Any signal in the green channel which is not colocalized with a red and/or blue signal is considered a false positive. These false positives are represented by the "other" category in figure 2 (<10% for all samples).

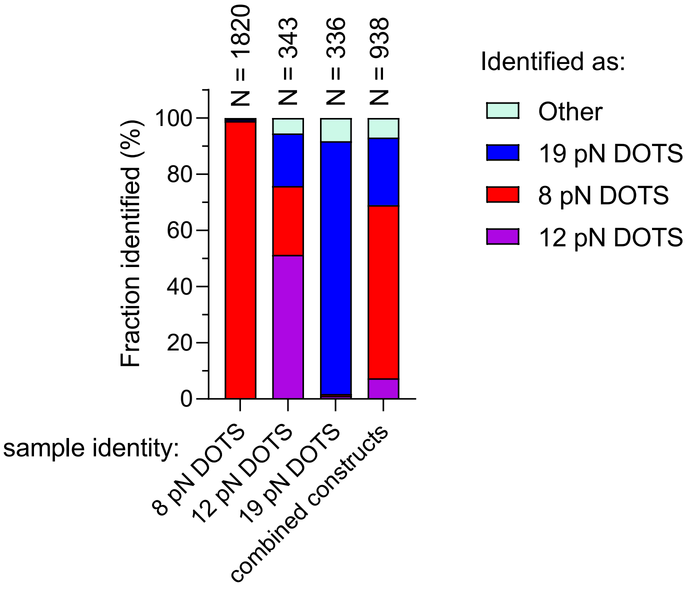

II. **Spatially localized under a T cell** – This criteria was added to ensure force generation signal was biologically relevant. Cy3B signal must occur under a cell to indicate a force event. Cy3B signal occurring in the background (away from cells) are considered false

positives. Given this criteria, force events represent ~20% of the population under cells, while false positives represent ~5% of the background population.

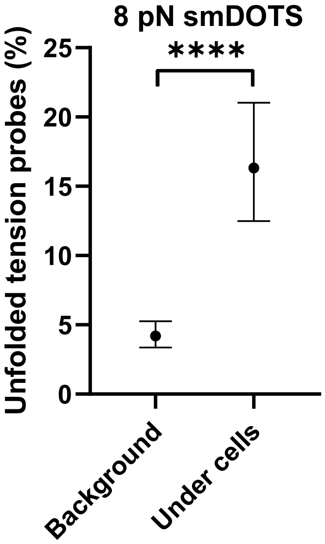

III. **The signal is intensity thresholded to minimize false positives.** The signal was calibrated using single-color controls to ensure >95% true positive rates while minimizing background.

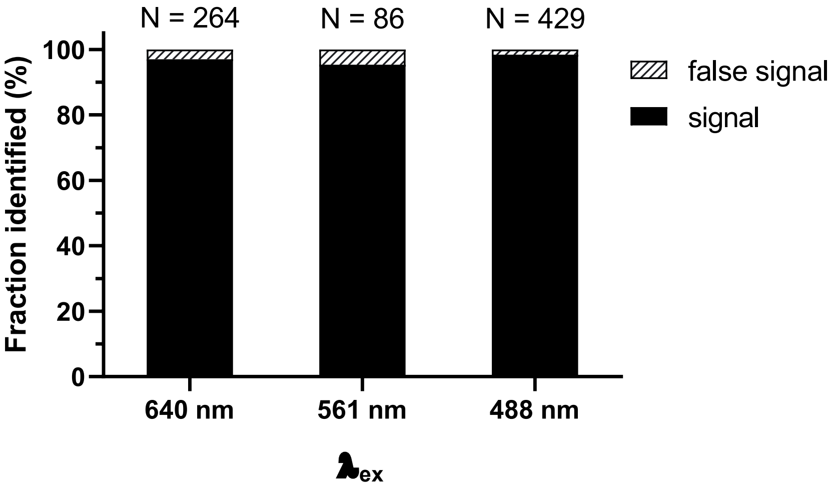

IV. **Bleedthrough correction.** As confirmed through single-color imaging with <2% observed cross-channel signal across three replicates. These colocalizations were later eliminated by intensity thresholding.

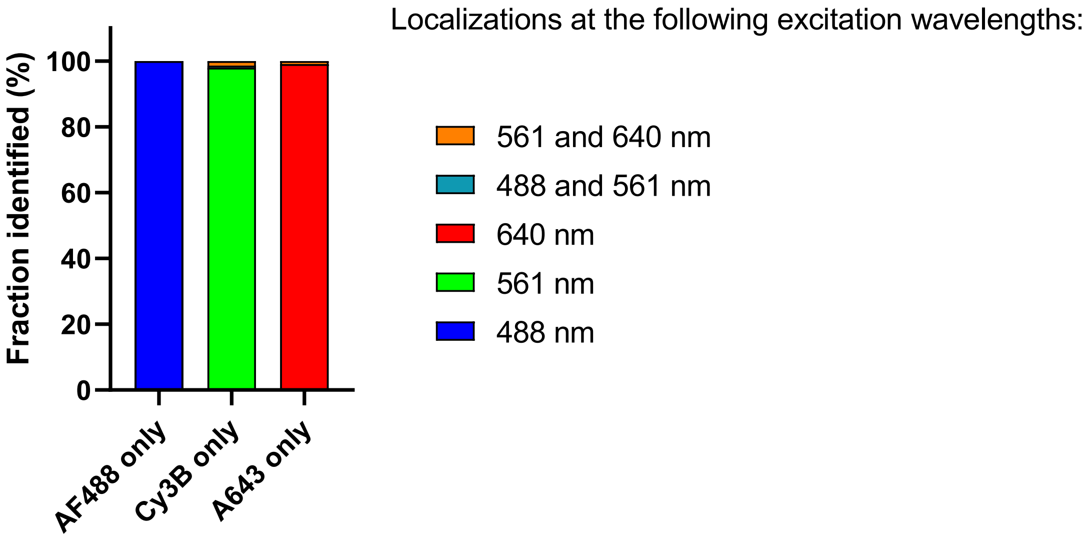

V. **Displayed appropriate diffusion coefficient**, with fast-diffusing particles excluded based on qualitative assessment.

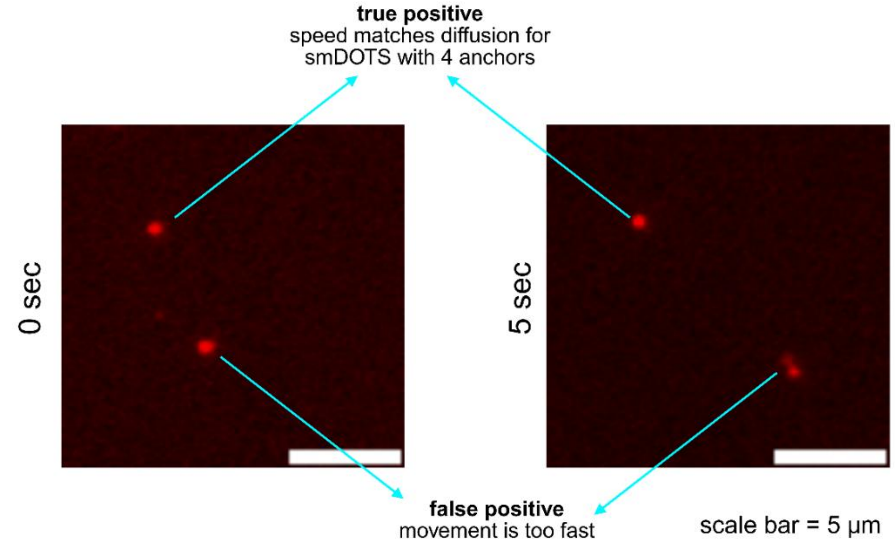

Together, these filters provide high confidence in our identification of TCR-transmitted force events. While labeling inefficiencies limit the total number of analyzable particles, they do not undermine the validity of the force events we do detect. After applying the above filtering criteria, the estimated probability that a detected signal is a true positive is >99% based on Bayesian logic. Summarized below are the true positive rates (TPR) and likelihood ratio (LR) for each probe based on the criteria described prior. LR is calculated as a ratio of true positives to false positives (FPR) for each criterion, with the combined LR being a product of the ratios. Posterior probability was calculated as the product of prior odds and LR. Here we assumed a conservative prior of 0.1, meaning that only 10% of detections are expected to be true positives. Note that for these calculations we excluded the last two criteria as they are difficult to quantify, but these further reduce FPR which further increases our confidence.

Probe	Fingerprint TPR	Channels Used	Intensity TPR	Combined LR	Posterior TP Probability
**8 pN**	98.6%	Red	97%	**~377,000**	**> 99.998%**
**12 pN**	51.3%	Red + Blue	97% × 98% = 95.1%	**~31,300**	**> 99.9968%**
**19 pN**	89.9%	Blue	98%	**~344,000**	**> 99.9985%**

The high confidence is a result of combining strong, mostly independent criteria with high specificity thresholds to eliminate any signal that potentially represents false positives. That said, we recognize that modeling assumptions may lead to overconfidence. To address this concern, we performed a sensitivity analysis to evaluate how deviations in input parameters affect our model's predictions. Specifically, we examined how the posterior probability of a true positive signal changes when we vary the true positive rate (TPR) and false positive rate (FPR) associated with the colocalization criterion, which is a key filter in our classification scheme. For each probe (8 pN, 12 pN, and 19 pN), we varied the colocalization TPR and FPR independently over a ±20% range from their nominal values while holding the other criteria (localization under a cell and intensity thresholding) constant. We then recalculated the Bayesian posterior probability for all combinations of these perturbed values. We found that even under conservative assumptions, for example increasing the FPR or decreasing the TPR beyond their estimated bounds, the posterior probability of a true positive remained consistently high. Even with 20% variation in both directions, confidence mostly stays above 85% for the 12 pN probe, 95% for the 19 pN probe, and 98.5% for the 8 pN probe. These calculations were conducted with the aid of ChatGPT[11].

3. Supplementary Tables and Figures

3.1. Supplementary Tables

Table 3.1.1. Modified DNA sequences used for smDOTS

Start 5'	End 3'	Note	Sequence 5’→3’	Conjugation
6[198]	8[199]	Additional Quencher	ACGGAACTGACGAGAAACA CCAGCGGTGTACA/3AmMO/	BHQ2-NHS ester
2[198]	4[199]	Additional Quencher	CTGAATAAGGAAGCCCGAA AGACATTGAATCC/3AmMO/	BHQ2-NHS ester
8[102]	10[103]	Density reporter	TGATACCCGATAAAGACGG AGGAAAATTGTTA/3AmMO /	A643-NHS ester *OR* DBCO-NHS ester followed by AF488-azide
8[70]	10[71]	Density reporter	GCAACTCACAGGGCTTAAG CTACTACGAGCCG/3AmMO /	A643-NHS ester *OR* DBCO-NHS ester followed by AF488-azide
4[102]	6[103]	Density reporter	GGGAACAAGCGCCATTCGC CATTCCAGCTGGC/3AmMO /	A643-NHS ester *OR* DBCO-NHS ester followed by AF488-azide
4[70]	6[71]	Density reporter	AGG TCA CCG GCA CCG CTT CTG GTG ATT AAG TT/3AmMO /	A643-NHS ester *OR* DBCO-NHS ester followed by AF488-azide
4[198]	6[199]	Staple + hairpin 8 pN hairpin	CCC TCA AAC ACT ATC ATA ACC CTA ACG AAC TA/iAmMC6T/ TTT GTA TAA ATG TTT TTT TCA TTT ATA CTT TGT GTC GTG CCT CCG TGC TGT G	BHQ2-NHS ester
4[198]	6[199]	Staple + hairpin 12 pN hairpin	CCC TCA AAC ACT ATC ATA ACC CTA ACG AAC TA/iAmMC6T/ TTT GTA CGC GCG TTT TTT TCG CGC GTA CGT GCC TCC GTG CTG TG	BHQ2-NHS ester
4[198]	6[199]	Staple + hairpin 19 pN hairpin	CCC TCA AAC ACT ATC ATA ACC CTA ACG AAC TA/iAmMC6T/ TTT GCG CGC GCG CGC TTT TGC GCG CGC GCG CGT GCC TCC GTG CTG TG	BHQ2-NHS ester

12[215]	10[216]	legs for SLB anchoring	CAA GCA GGC GAG CGG ACG AAC TTT TCTCCAAACGCTGAGGCTT GCAGGGACTTTTT	
12[55]	10[56]	legs for SLB anchoring	CAA GCA GGC GAG CGG ACG AAC TTT AGCAGGCGGAAACCTGTCG TGCCAAAAGTGTA	
4[183]	2[184]	legs for SLB anchoring	CAA GCA GGC GAG CGG ACG AAC TTT AACAGTTCCATCAAAAAGA TTAAGTAATGCTG	
4[55]	2[56]	legs for SLB anchoring	CAA GCA GGC GAG CGG ACG AAC TTT AGATGGGCTTTTGTTAAATC AGCTAAAGCCCC	
12[87]	10[88]	legs for SLB anchoring	CAA GCA GGC GAG CGG ACG AACTTT GTTGCAGCGGCCAACGCGC GGGGAACAATTCC	
12[183]	10[184]	legs for SLB anchoring	CAA GCA GGC GAG CGG ACG AAC TTT AATTGTATACCATCGCCCAC GCATGTAAAATA	
4[215]	2[216]	legs for SLB anchoring	CAA GCA GGC GAG CGG ACG AAC TTT AAATATTCTTCAAATATCGC GTTTTTGCGGAT	
4[87]	2[88]	legs for SLB anchoring	CAA GCA GGC GAG CGG ACG AAC TTT ATTGACCGGGAACGCCATC AAAAAGTCAATCA	
		Top strand	/5Biosg/CA CAG CAC GGA GGC ACG ACA C/3AmMO/	Cy3B-NHS ester
		cholesterol anchor	GTT CGT CCG CTC GCC TGC TTG /3CholTEG/	

Table 3.1.2. Masses of staple strands labeled by spectral fingerprint dye

Staple strand/modification	Expected mass (Da)	Measured mass (Da)
8[70]-10[71]/A643	10832	10831
8[102]-10[103]/A643	10958	10958
4[70]-6[71]/A643	10860	10860
4[102]-6[103]/A643	10824	10825
8[70]-10[71]/AF488	10994	10994
8[102]-10[103]/AF488	11120	11121
4[70]-6[71]/AF488	11022	11022
4[102]-6[103]/AF488	10986	10985

Table 3.1.3. List of 77 unmodified DNA staple sequences

Start 5'	End 3'	Sequence 5'→ 3'
4[38]	6[39]	CCGTGCAGCCTCAGGAAGATCGCTCACGACGT
8[215]	6[216]	CAGATGAAAACGAGTAGTAAATTGAAATCTAC
10[166]	12[167]	GGCACCAGCCGACAATGACAACACGGTTTATC
7[128]	7[159]	AATAACCCCGCCATTACCCAAATCAACGTAAC
0[247]	2[231]	GCATTAACATCCAATAAATCATACATAACCTGTTTAGCTATGATAAGAG
12[198]	12[216]	CCAAAAGTTGTCGTCTTTCCAGACGTTAGTAAATGAATTGTTGAAAA
1[128]	1[159]	TGAGAGTCTGGAGTTTCATTCCATATAACAGT
12[230]	12[248]	TTTTCACTTCTGTATGGGATTTTGCTAAACAACTTTCAAACTAAAGG
2[38]	4[39]	ATAAGCAAAAATTCGCATTAAATGCATCGTAA
10[70]	12[71]	GAAGCATGCTGCATTAATGAATCAAGCGGTCC
10[215]	8[216]	CATGAGGAATTTGTATCATCGCCTAAAGAGGA
4[119]	2[120]	CCCGTCGGTTCCTGTAGCCAGCTTGAATCGAT
0[215]	2[199]	CAAAGAATTAGCAAAATTAAGCAATTTGACCATTAGATACGCTTAATTG
10[87]	8[88]	ACACAACAGTGGTGCTTGTTACCTGACAGTGC
13[128]	13[159]	ACCGTCTATCACGCCTGTAGCATTCCACAGAC
6[230]	8[231]	GGAAGAAGGCTTGAGATGGTTTAGAACTGACC
4[166]	6[167]	GAATGACGCCAAAAGGAATTACGGAAAGATTC
2[119]	0[120]	GAACGGTACTATCAGGTCATTGCCGCGGGAGA
8[55]	6[56]	GCACGAATTCTAAGTGGTTGTGAAGCCAGGGT
2[230]	4[231]	GTCATTTTAATTCGAGCTTCAAACGTCCAATA
8[198]	10[199]	GACCAGGACAAAGTACAACGGAGAGTTTCCAT
10[38]	12[39]	TGAGTGACCCGCTTTCCAGTCGGAAAATCCTG
12[119]	10[120]	CTGATTGCTGGGCGCCAGGGTGGTAGCTGTTT
0[87]	2[71]	TAGAACCCTCATATATTTTAAATGATAAATTAATGCCGGACCGGTTGAT
6[166]	8[167]	ATCAGTTAAAGCTGCTCATTCAGCCTTCATCA
6[119]	4[120]	CTATTACGCAGGCTGCGCAACTGTAGTAACAA
6[247]	4[248]	TACCAGTCCGAGAGGCTTTTGCAATGTTTAGA
6[159]	6[128]	GAGATTTAGGAATACCACATTATCGGTGCGGG
2[166]	4[167]	AATATGCGAAGCAAAGCGGATTGAGAAAACGA
8[87]	6[88]	GGCCCTGCAAGTGTCCTTAGTGCTGGATGTGC
6[183]	4[184]	TACAGGTAAGGCATAGTAAGAGCAATGCTTTA
6[55]	4[56]	TTTCCCAGACTCCAGCCAGCTTTCGTTGGTGT
2[87]	0[88]	TATGTACCGAGGGTAGCTATTTTTAAAATTTT
9[128]	9[159]	TCGAATTCGTAAGAATACACTAAAACACTCAT

0[183]	2[167]	CAGAGCATAAAGCTAAATCGGTTGTGATTCCCAATTCTGCCATG TTTTA
6[70]	8[71]	GGGTAACTTCATGCGCACGACTTCATCTGTAA
8[166]	10[167]	AGAGTAACTTTGACCCCCAGCGACACTACGAA
8[38]	10[39]	TTGAATCAACTCTGACCTCCTGGGGTGCCTAA
10[198]	12[199]	TAAACGGAACCGATATATTCGGTAAAAAGGCT
10[247]	8[248]	GCTACAGAATCCGCGACCTGCTCCCAATCATA
12[70]	12[88]	ACGCTGGTGTTCCAGTTTGGAACAAGAGTCCACTATTAACTGAG AGA
6[215]	4[216]	GTTAATAACGTTTACCAGACGACGATCGTCAT
10[119]	8[120]	CCTGTGTGTCCCCGGGTACCGAGCTTACGCTC
10[230]	12[231]	GACTAAAGAGTTAAAGGCCGCTTATAATAATT
3[128]	3[159]	TCATCAACATTTTACCCTGACTATTATAGTCA
0[55]	2[39]	GAGTAATGTGTAGGTAAAGATTCAACCATCAATATGATATGAAG ATTGT
10[159]	10[128]	ACCTAAAACGAAAGAGGCAAAATCATGGTCAT
5[128]	5[159]	TGGGAAGGGCGCAACTAATGCAGATACATAAC
4[159]	4[128]	CATAAATCAAAAATCAGGTCTAAATGTGAGCG
10[183]	8[184]	CGTAATGCTTATACCAAGCGCGAACGCATAGG
0[119]	2[103]	AGCCTTTATTTCAACGCAAGGATAGAGAGATCTACAAAGGATCG TAAAA
12[102]	12[120]	CCTGGCCAGAACGTGGACTCCAACGTCAAAGGGCGAAAAGGCA ACAG
4[230]	6[231]	CTGCGGAATAAAAACCAAAATAGAGGACGTTG
2[247]	0[248]	GCTCCTTTTATTTTCATTTGGGGCTAGTAGTA
4[247]	2[248]	CTGGATAGGCGAACCAGACCGGAACTTTAATT
8[247]	6[248]	AGGGAACCATTTCAACTTTAATCAGCTCATTA
10[102]	12[103]	TCCGCTCGAGGCGGTTTGCGTATCCTTCACCG
6[87]	4[88]	TGCAAGGCGCCGGAAACCAGGCAAAACGGCGG
8[119]	6[120]	GCCCTGGAGACAATGTCCCGCCAACCTCTTCG
11[128]	11[159]	TTTTCTTTTCACAGCTTGATACCGATAGTTGC
8[159]	8[128]	TCTTGACAAGAACCGGATATTTTCTAATCTAT
6[102]	8[103]	GAAAGGGGAATTGTCAACCTTATGTGACTCTA
12[38]	12[56]	TTTGATGTCAAAAGAATAGCCCGAGATAGGGTTGAGTGTTTTGC CCC
2[215]	0[216]	GGCTTAGAATTTCGCAAATGGTCAAGGCAAGG
12[166]	12[184]	AGCTTGCAGCCCTCATAGTTAGCGTAACGATCTAAAGTTGAGCC TTT
6[38]	8[39]	TGTAAAACCAGGGTGGATGTTCTATAGGGGCC
8[183]	6[184]	CTGGCTGATGAATAAGGCTTGCCCAACATTAT
2[159]	2[128]	AACTAAAGTACGGTGTCTGGAAGCAAACAAGA
12[247]	10[248]	AATTGCGATTGCGGGATCGTCACCTAGCAACG
2[183]	0[184]	TAGCTCAAGAACGAGTAGATTTAGTAAAGCCT
8[230]	10[231]	AACTTTGGATAAATTGTGTCGAAGGCTTTGAG

12[159]	12[128]	TTTCGAGGTGAATTTCTTAAACCAGTGAGACG
2[55]	0[56]	AAAAACAGTCAACCGTTCTAGCTGCAATGCCT
0[159]	0[128]	TACCAAAAACATTATGACCCTGTAATACTTTT
2[102]	4[103]	CTAGCATTAATTCGCGTCTGGCCATTCTCCGT
10[55]	8[56]	AAGCCTGGTTGGTGTAATGAGTAAGTCGGTGG
2[70]	4[71]	AATCAGACATTTTTTAACCAATATAATGGGAT

Table 3.1.4. Optical configuration for imaging channels

**Color**	Red	Green	Blue
**Associated dye**	Atto 643	Cy3B	AlexaFluor 488
**Purpose**	Spectral fingerprinting	Tension indicator	Spectral fingerprinting
**Excitation wavelength (nm)**	640	561	488
**Emission wavelength (nm)**	675-775	580-630	500-550
**EM gain**	200	200	300
**Exposure time (ms)**	50	50	50
**Frame rate (Hz)**	0.0167	0.0167	0.0167
**Laser power (%)**	10	10	10
**Concurrent channel**	Green	Red	None
**Detector**	Cam. 1	Cam. 2	Cam. 2
**Electrons per A/D count**	16.5	15.1	15.1
**Single pixel noise**	179	166	166
**Peak intensity threshold**	std(Wave.F1)*2	std(Wave.F1)*2	std(Wave.F1)*1.3

3.2. Supplementary Figures

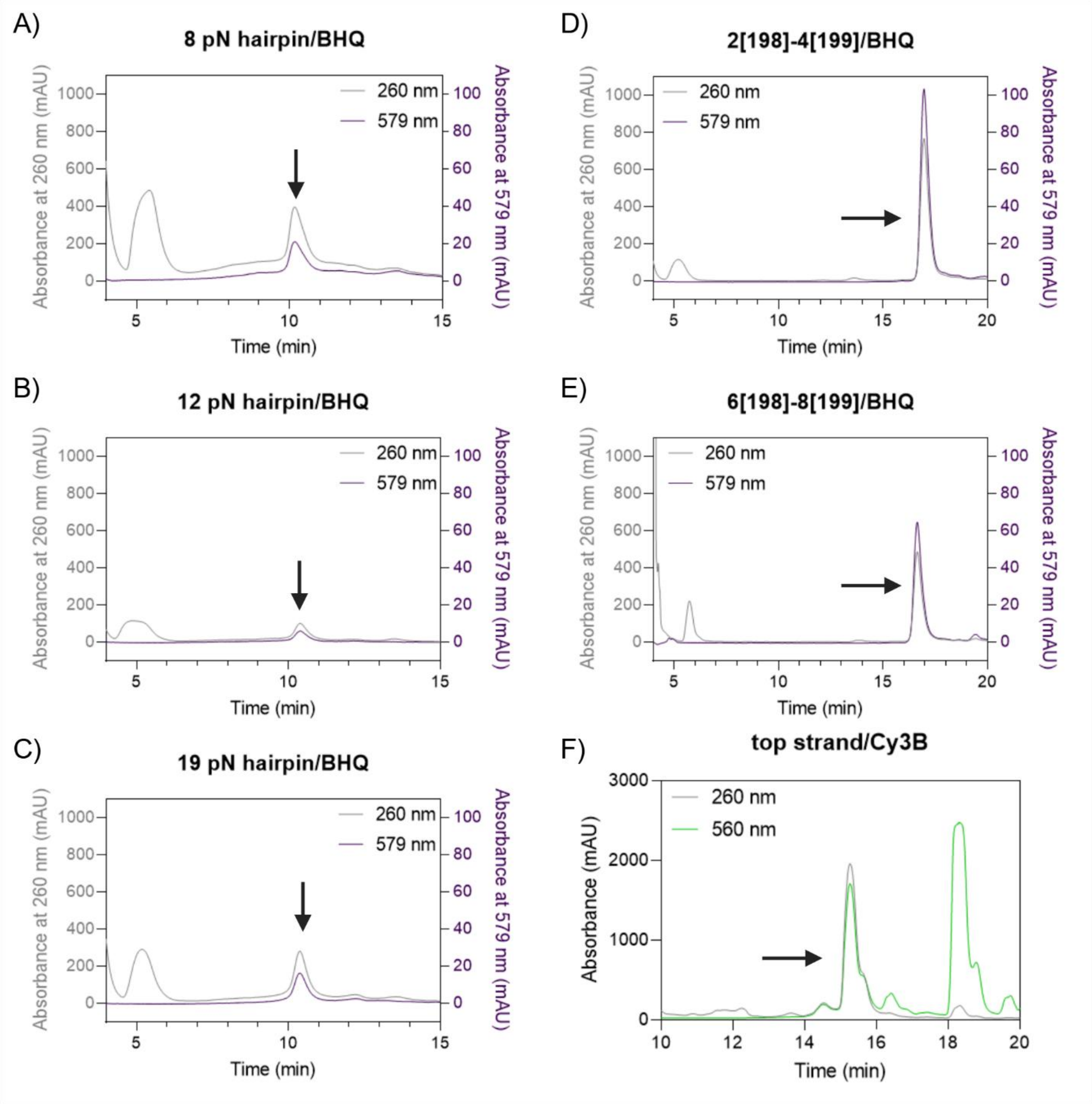

Figure 3.2.1. HPLC chromatograms for BHQ modified staple strands and Cy3B labeled top strand. A-C represent three hairpins with varying force thresholds. Each is modified with a fluorescence quencher. D and E represent staple strands modified with additional quenchers at 5 nm distance from the hairpins. F shows the top strand which binds to the hairpins, modified with

Cy3B which generates a fluorescence signal upon force-induced unfolding. Arrows indicate the product peaks which were collected and used for origami assembly.

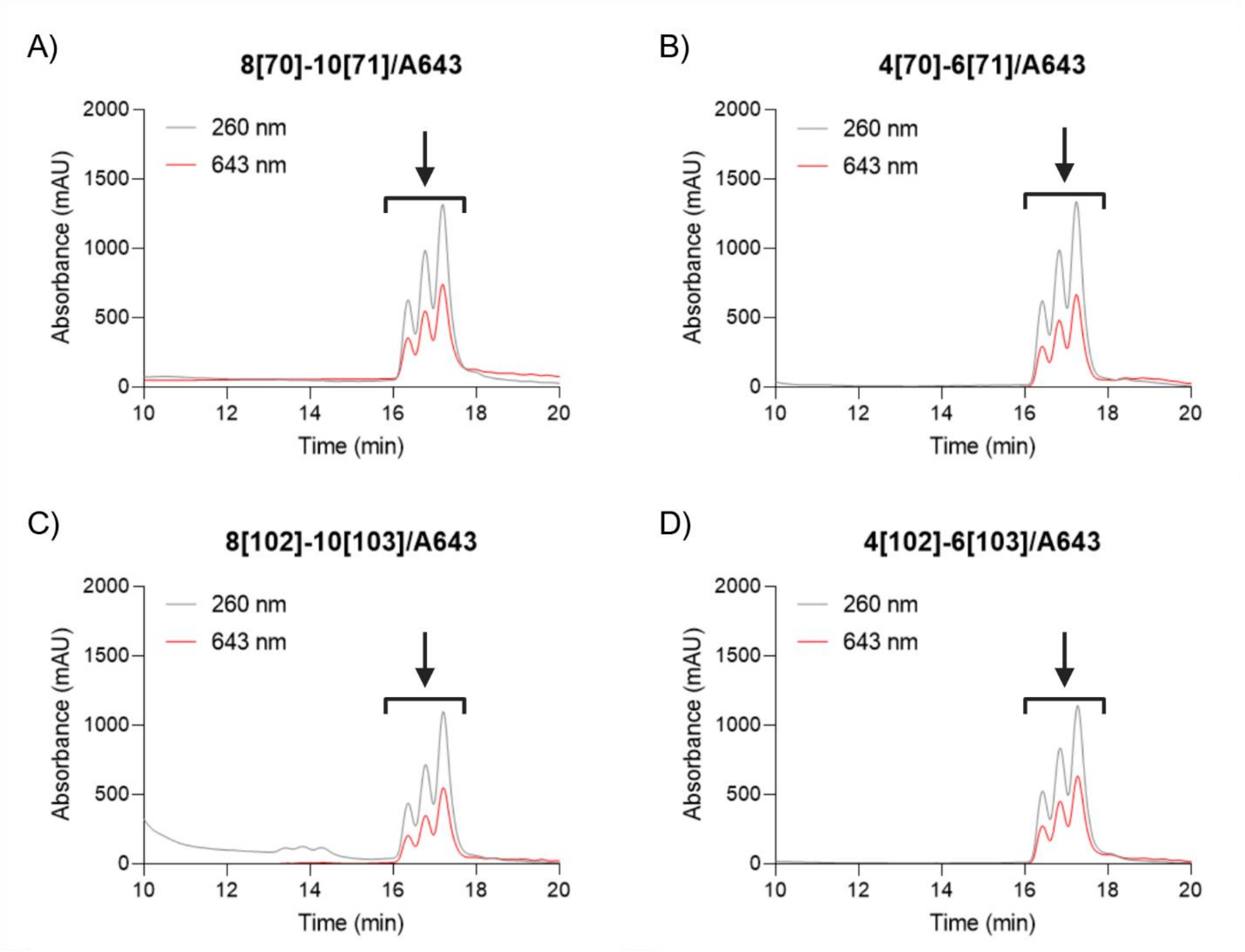

Figure 3.2.2. HPLC chromatograms for Atto 643 labeled staple strands. Four staple strands modified with Atto 643 to be used as density reporters/spectral fingerprints. Arrows indicate the product peaks which were collected and used for origami assembly. The three peaks shown in each chromatogram represent the three isomers of Atto 643[12]. All three peaks were collected and verified by mass spectrometry.

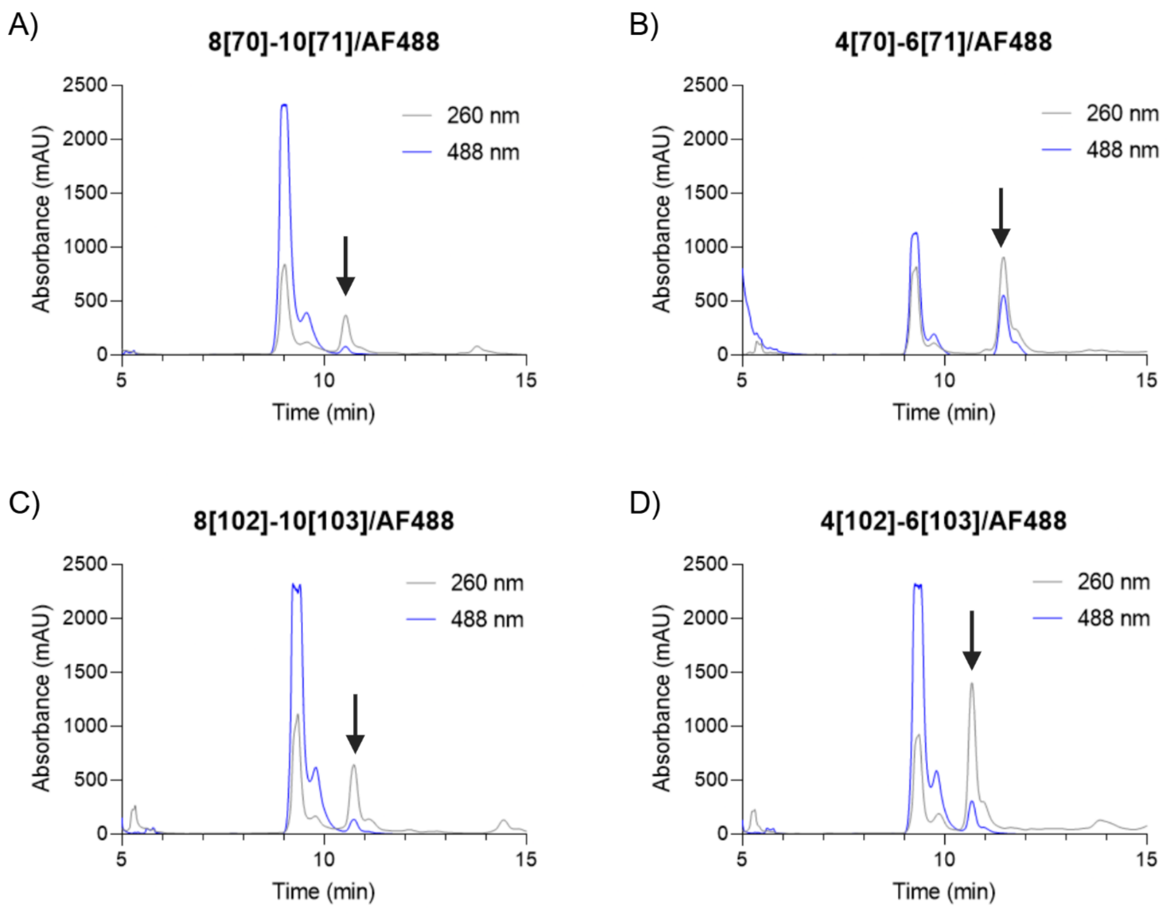

Figure 3.2.3. HPLC chromatograms for AlexaFluor 488 labeled staple strands. Four staple strands modified with AlexaFluor 488 to be used as density reporters/spectral fingerprints. Arrows indicate the product peaks which were collected and used for origami assembly.

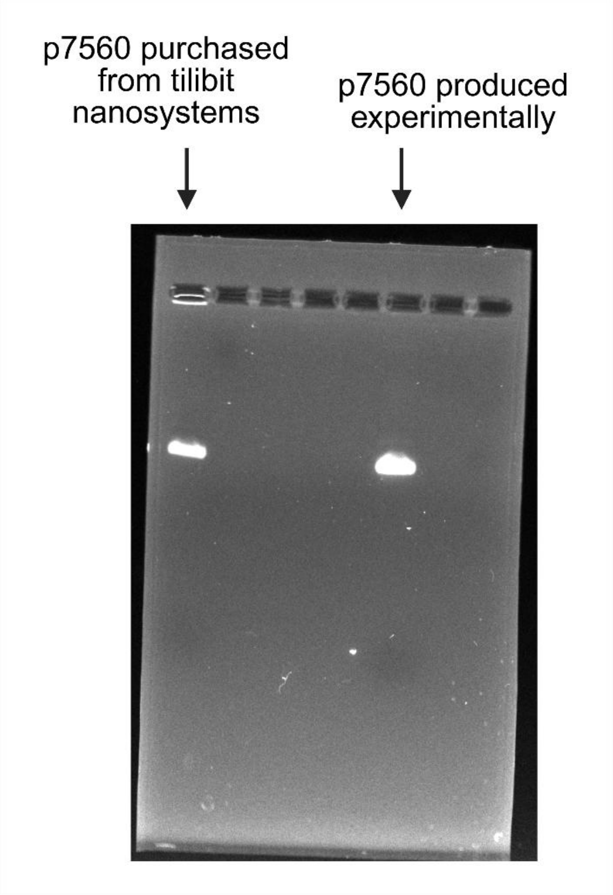

Figure 3.2.4. Agarose gel characterization of origami scaffold strand. Left: p7560 single stranded DNA origami scaffold purchased from tilibit nanosystems. Right: p7560 single stranded DNA origami scaffold produced experimentally for this work.

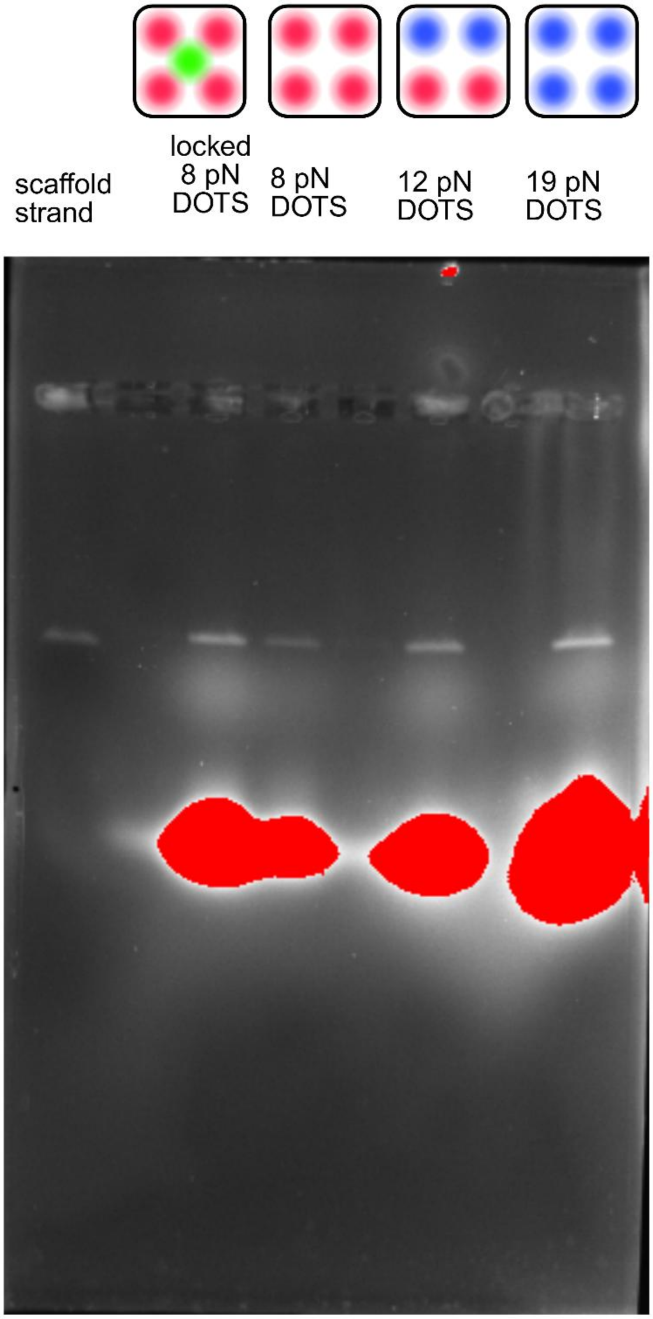

Figure 3.2.5. Full gel images representing ethidium bromide staining of nucleic acids. Red signal represents overexposure due to high concentrations of staple strands.

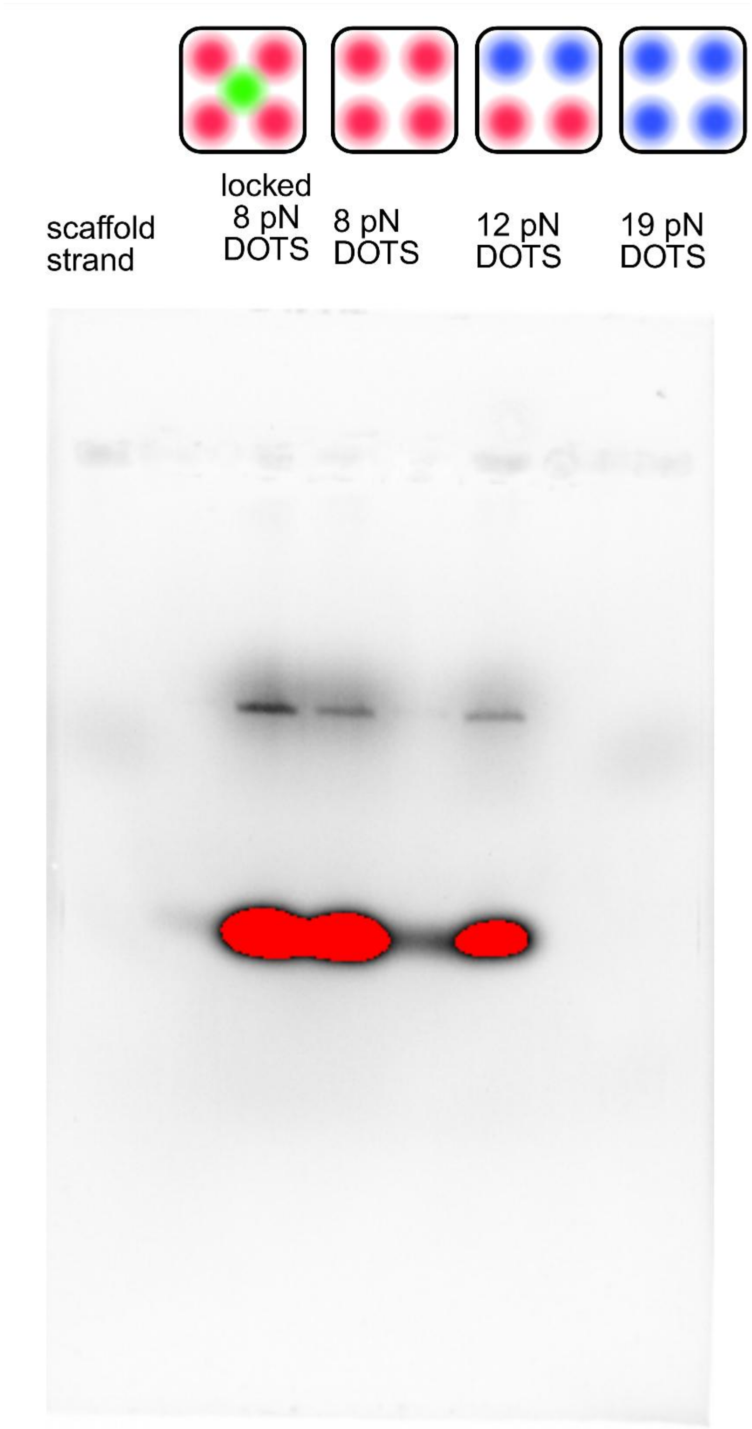

Figure 3.2.6. Full gel images representing Atto 643 dye labeling. Red signal represents overexposure due to high concentrations of staple strands.

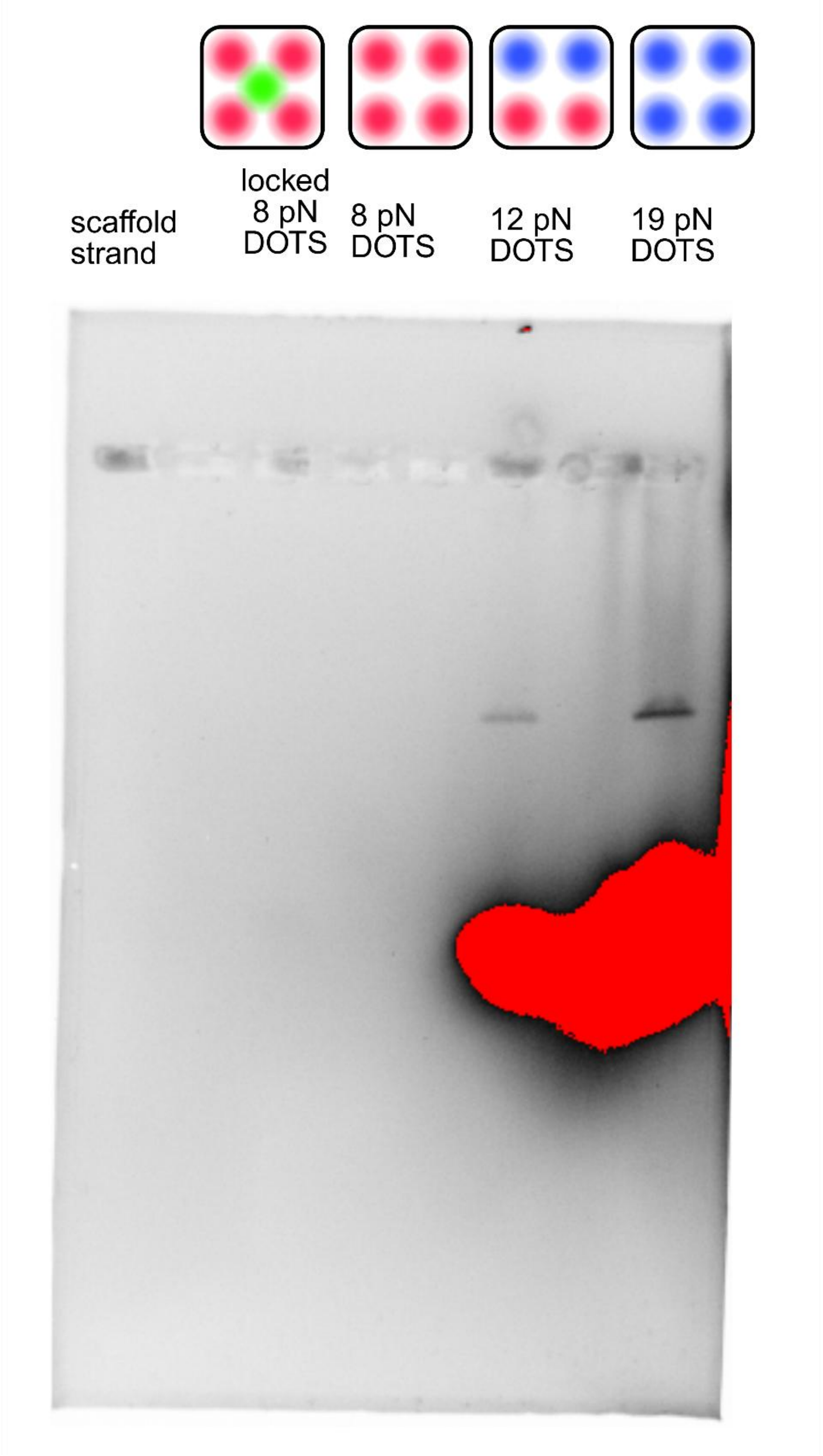

Figure 3.2.7. Full gel images representing AF488 dye labeling. Red signal represents overexposure due to high concentrations of staple strands.

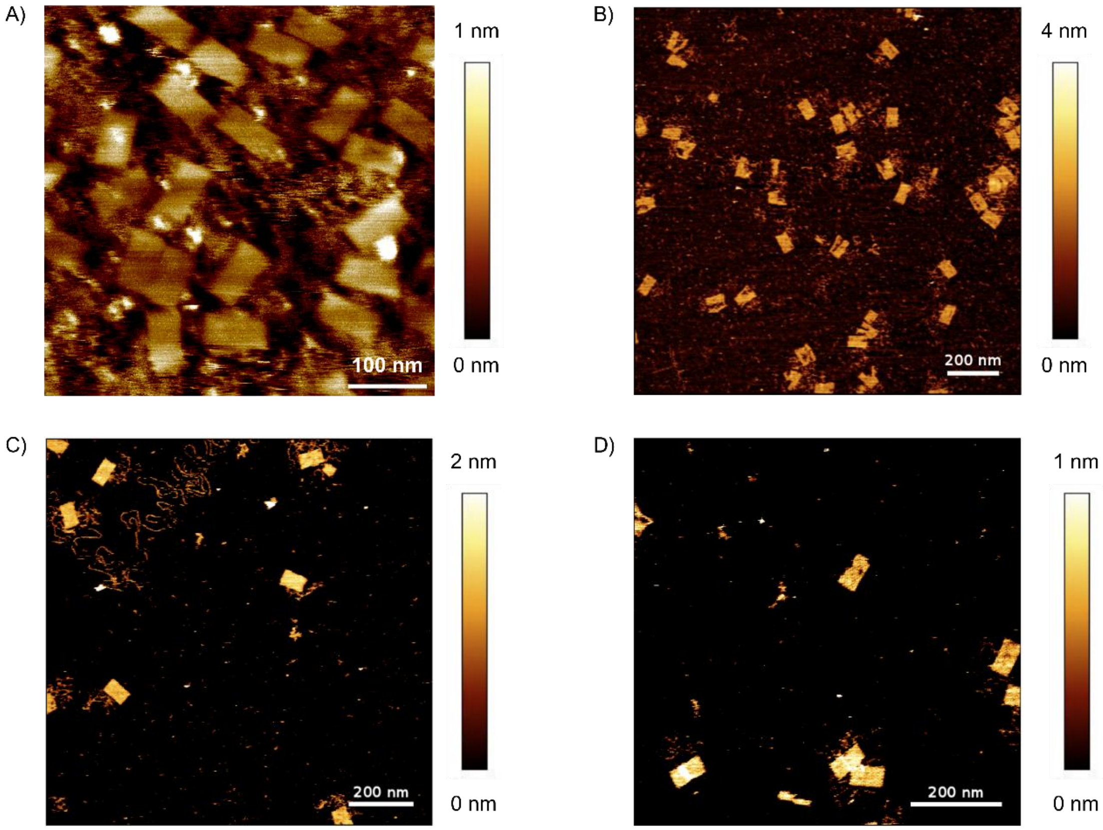

Figure 3.2.8. AFM images of multiple smDOTS structures. A) This image was obtained immediately after synthesis using a Bruker Multimode 8 AFM and shows ~20 fully intact DNA nanostructures. B, C, D) smDOTS constructs were shipped to Bruker for higher quality imaging. These samples were imaged ~3 weeks after synthesis, leading to partial degradation in some of the structures.

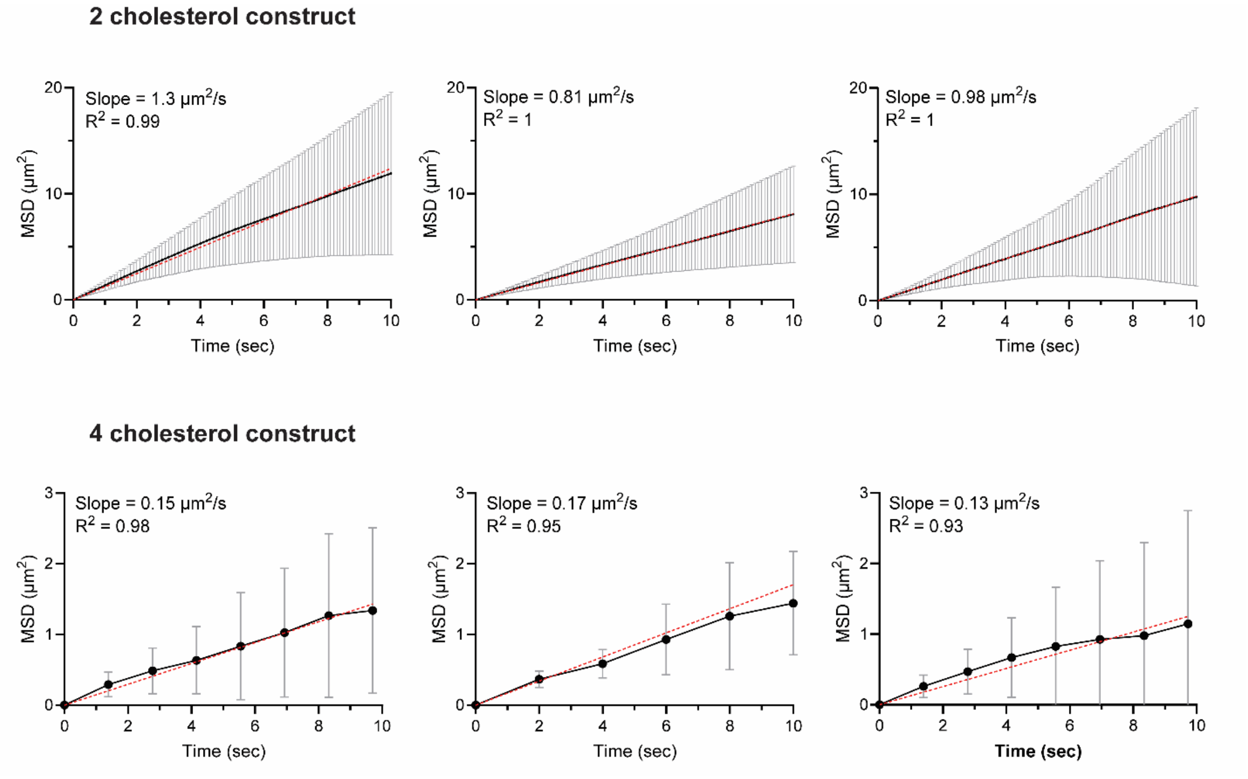

Figure 3.2.9. Diffusion rates of smDOTS measured from three independent replicates. These plots represent MSD vs. time for smDOTS with 2 cholesterol anchors (top) and 4 cholesterol anchors (bottom). Particle localizations were identified using THUNDERSTORM as previously described. Those localizations were clustered into tracks using dbscan, stationary tracks were removed, and the mobile tracks were processed using MSDanalyzer[13]. The dashed red line represents the linear fit, the slope of which is proportional to the diffusion rate. A linear plot indicates Brownian motion. Error bars represent the margin of error from upper and lower bounds.

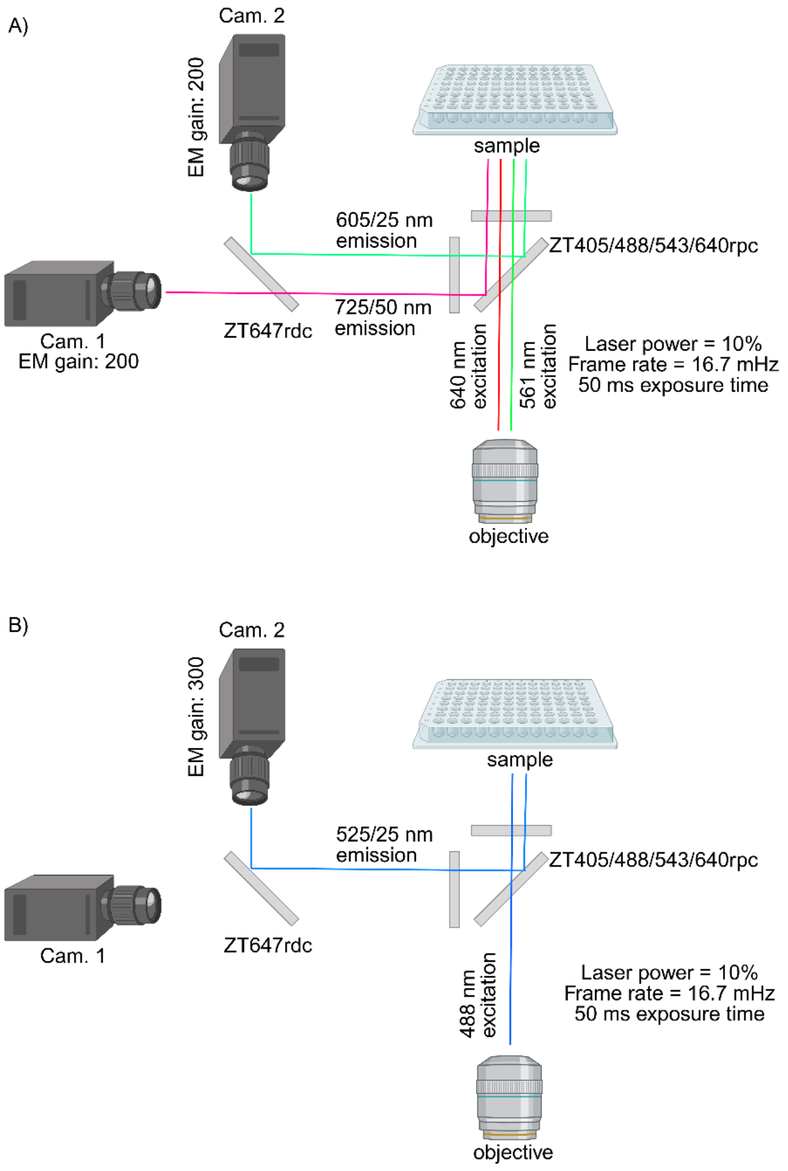

Figure 3.2.10. Optical configuration for fluorescence imaging. A) Atto 643 and Cy3B are imaged simultaneously with two active lasers at 640 nm and 561 nm excitation, respectively. Emitted light is split using a long pass dichroic mirror at 647 nm, with shorter and longer wavelengths detected by two separate cameras. B) Afterwards, AlexaFluor 488 is imaged independently using a 488 nm excitation laser. Note: Cy3B was used to indicate tension, while Atto 643 and AF488 were used as spectral fingerprints to identify particles.

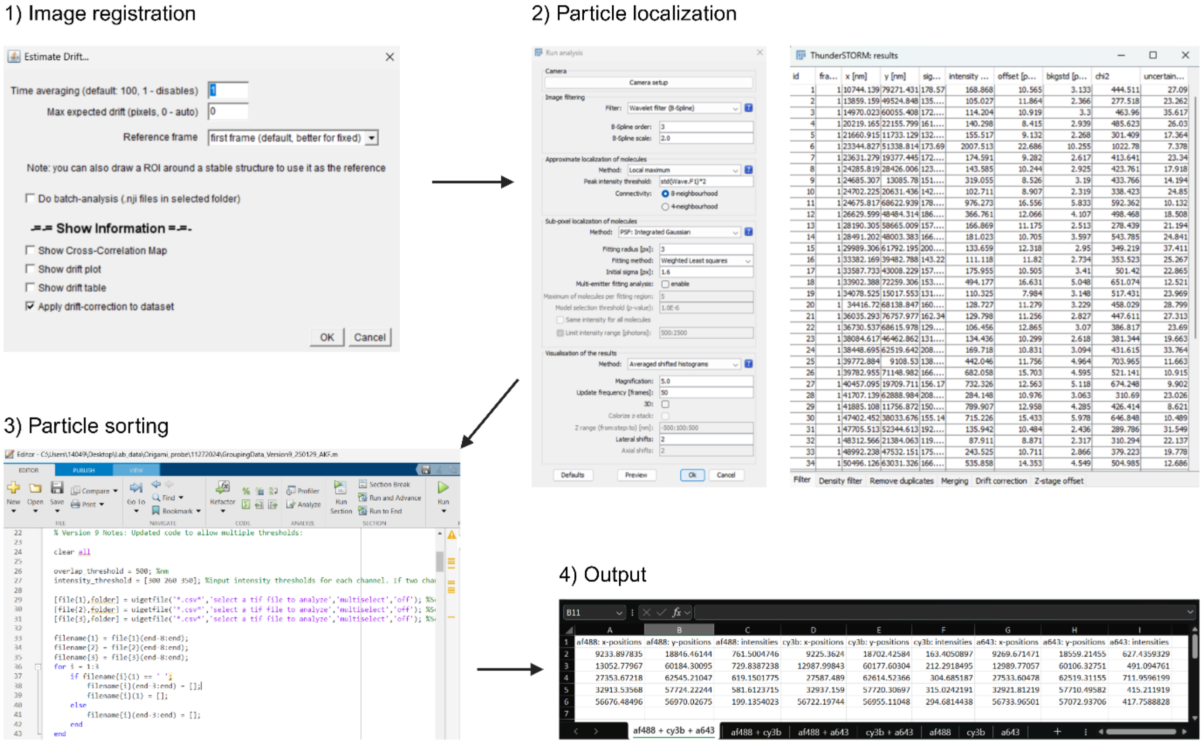

Figure 3.2.11. Flowchart of data analysis workflow. First, images were registered across different channels using Fast4Dreg. Then, particles were identified in each channel using Thunderstorm. Afterwards, particles were sorted based on the channels they appeared in using a custom Matlab algorithm to produce spreadsheets detailing the location and intensity of particles in each channel combination.

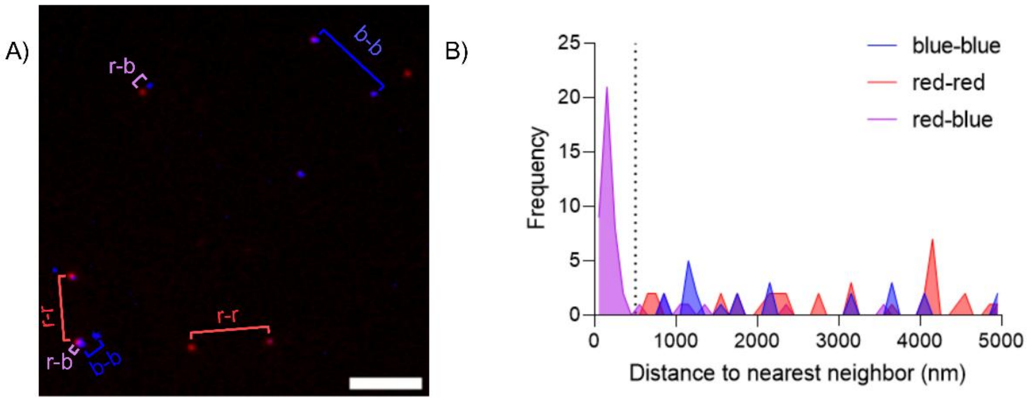

Figure 3.2.12. Determination of nearest-neighbor distances for identification of colocalized particles. A) Representative composite image showing 12 pN smDOTS particles presenting A643 and/or AF488 dyes. Scale bar = 5 µm. Distances between each particle and its nearest neighbor were measured between two red particles (red-red), two blue particles (blue-blue), or one red and one blue particle (red-blue). These distances were plotted in the histogram shown in B). This histogram was used to determine the overlap threshold for identifying particles in more than one channel. Particles presenting both a red and a blue dye have a red-blue distance < 500 nm, while red-red distances and red-blue distances exceed 500 nm.

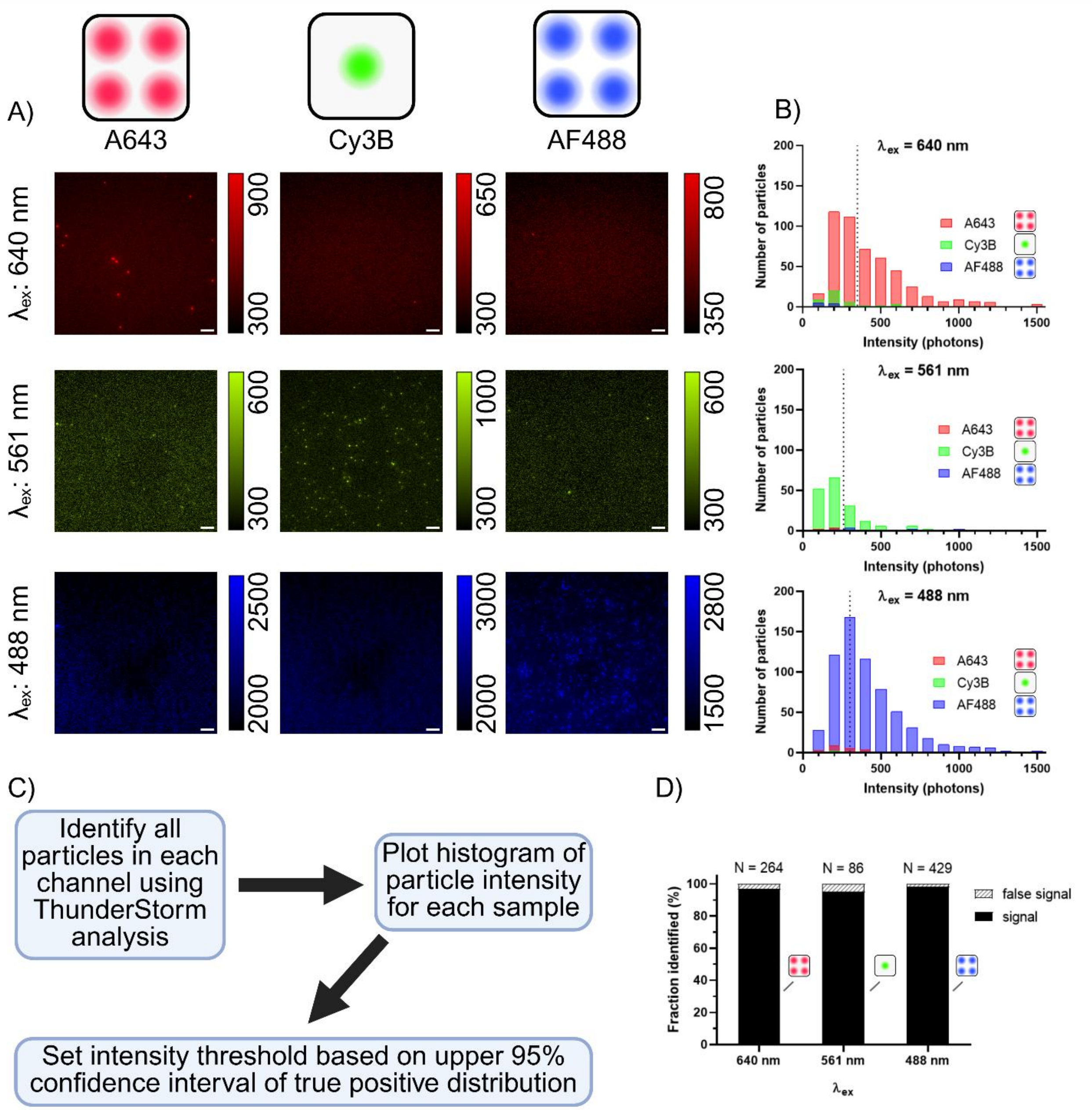

Figure 3.2.13. Quantification of bleedthrough and noise across three fluorescence channels. A) Columns: representative images of smDOTS constructs, each containing one dye only: Atto 643, Cy3B, or AlexaFluor 488 (left to right). Rows: images were obtained in three fluorescence channels, each corresponding to one of the fluorophores: 640 nm for A643, 561 nm for Cy3B, and 488 nm for AF488 (top to bottom). Scale bar = 5 μm. B) For each channel, a histogram was plotted

showing the number of particles identified and their intensities within a given sample. The dotted lines represent the cutoff values used for intensity thresholds: 300 for the AF488 channel, 260 for Cy3B, and 350 for the A643 channel. Data was pooled from three replicates. C) Overview of workflow used in determining bleedthrough rates and setting intensity thresholds. D) Comparison of the accuracy of particle identification before and after thresholding. Here, “signal” represents the number of particles in samples which contain the organic dye corresponding to the excitation wavelength (A643 for 640 nm, Cy3B for 561 nm, and AF488 for 488 nm). Samples which show signal but do not contain the appropriate dye for a given channel (Cy3B and AF488 for 640 nm, A643 and AF488 for 561 nm, A643 and Cy3B for 488 nm) are labeled as “false signal”. After thresholding, we were able to identify particles in each channel with >95% accuracy.

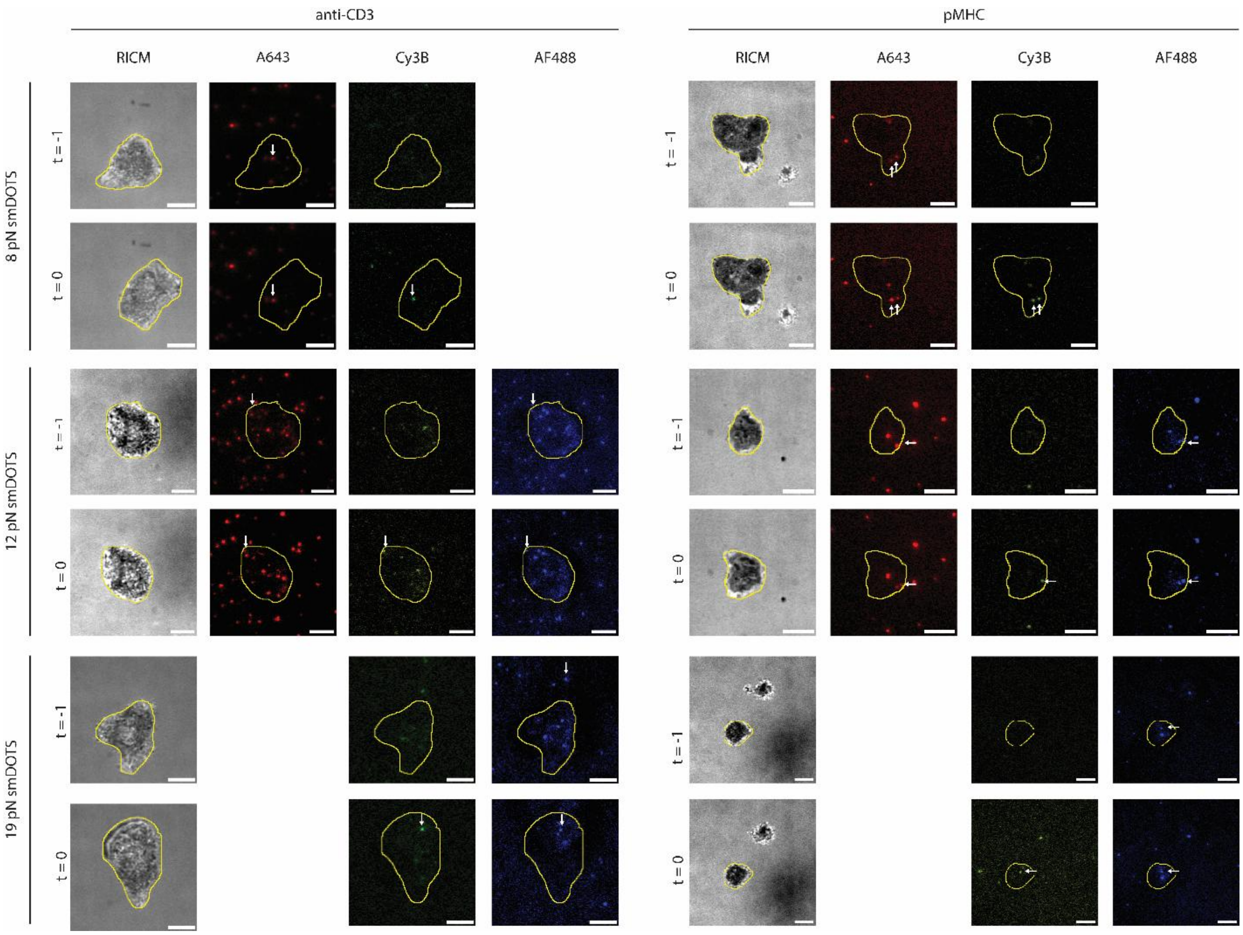

Figure 3.2.14. Additional examples of tension measurements. Additional examples are provided for each probe (8 pN, 12 pN, and 19 pN) using two different biological ligands (anti-CD3, pMHC) to demonstrate reproducibility of tension measurements. Time points (min) are centered on the observation of force. White arrows indicate particles of interest in each channel: spectral fingerprinting channels at all time points, and Cy3B channel at force occurrence (t = 0). Scale bars = 5 μm.

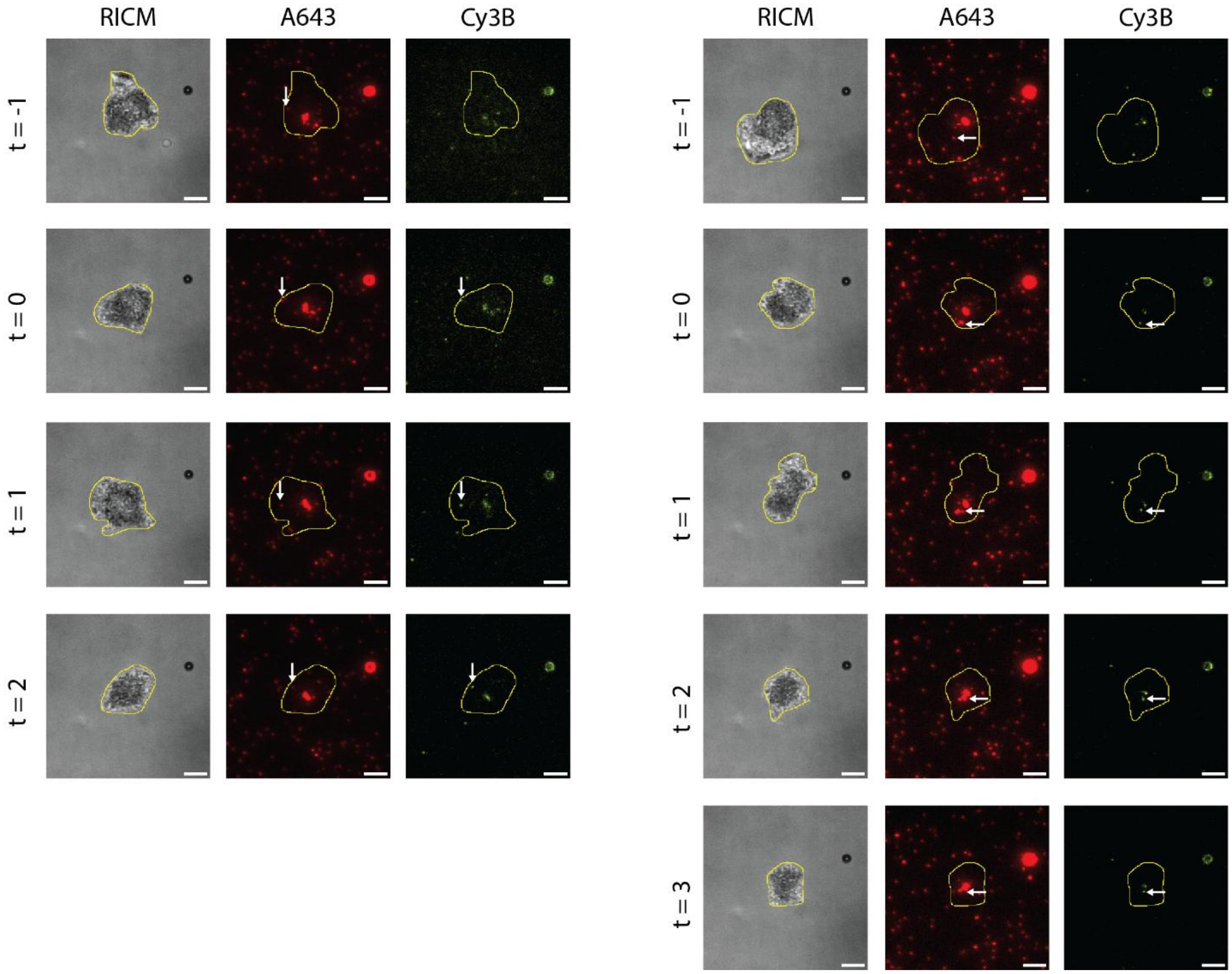

Figure 3.2.15. Additional examples of ligand translocation following force activation. Additional examples are provided to demonstrate reproducibility. Here, we see two separate instances of force events, followed by movement of the probe towards an apparent cluster near the center of the cell. Time points are centered on the observation of force (t = 0 min). White arrows indicate particles of interest in each channel: spectral fingerprinting channel across all time points to indicate the probe translocation, and Cy3B channel at t = 0 onwards to show mechanical activity. Scale bars = 5 μm.

4. MATLAB scripts

4.1. Particle tracking algorithm

```
% This section loads in localization data from Thunderstorm addon:

[file_THUNDERSTORM,folder_THUNDERSTORM] = uigetfile('*.csv*','select a csv file to analyze');

% This section determines the number of files selected and defines them all as type cell NOTE: this is a generic method for converting file inputs into cell format (for syntax consistency). However, since this code always analyzes one run at a time nfile will always = 1 and need conversion to cell

if iscell(file_THUNDERSTORM)

    nfile = length(file_THUNDERSTORM); %count number of files

else

    nfile = 1; %if only one file format is different (saved as string vs cell)

    file_THUNDERSTORM = {file_THUNDERSTORM}; %convert to cell

end



% This section loads localizations and marks their frame

for i = 1:nfile

    [frame X Y] = csvimport([folder_THUNDERSTORM,file_THUNDERSTORM{i}], 'columns', {'"frame"','"x [nm]"', '"y [nm]"'},'ignoreWSpace',true);
```

```
end

% This section simply counts the number of events in each frame (indices):

clear indices

indices_count(max(frame)) = 0;

indices = cell(1,max(frame));

for i = 1:max(frame)

    indices_count(i) = length(find(frame == i));

    indices{i} = find(frame == i);

end


% % % % % % % % % % % % %% % % % % % % % % % % %% % % % % % % % % %

% % % % % % % % % % % % % % Calculating Diffusion!% % % % % % % % % % % % %

% % % % % % % % % % % % %% % % % % % % % % % % %% % % % % % % % % %


% This section uses Dbscan to create tracks from particle localizations:

epsilon = 1000; %nm - Distance particle can move in x/y and still be grouped

max_frame_dist = 2; %number of frames which can be missed and have the particle still be grouped
```

```
dbscan_weighting = [1,1,epsilon./(max_frame_dist+1)+1/(max_frame_dist+1)]; %Set frame to JUST over epsilon so that we will never get a trajectory from 2 frames away.
dbscan_minpts = 3;
start = clock;
clusters = dbscan([X,Y,frame].*dbscan_weighting,epsilon,dbscan_minpts);
finish = clock;
cluster_indices = cell(max(clusters),1);
clear cluster_length cluster_unique
cluster_length(max(clusters)) = 0;
cluster_unique(max(clusters)) = false;
for i = 1:max(clusters)
    indices = find(clusters==i);
    cluster_indices{i} = indices;
    cluster_length(i) = length(indices);
    if length(unique(frame(indices))) == length(frame(indices))
        cluster_unique(i) = true;
    end
end
```

```
long_track_indices = find(cluster_length>100); %Find long-lived tracks based on arbitrary frame count (for analyzing the code and for potential downstream analysis)

long_unique_track_indices = intersect(long_track_indices,find(cluster_unique));



% This section calculates diffusion based on previously found tracks

% This section of code was adapted from Ref. 7

% Define units and initialize analyzer:

SPACE_UNITS = 'nm';

TIME_UNITS = '1 sec';

ma = msdanalyzer(2, SPACE_UNITS, TIME_UNITS);




% Formulate tracks ([ Ti Xi Yi ...]):

tracks = cell(length(long_unique_track_indices),1);



for i = 1:length(long_unique_track_indices)

    tracks{i} = [frame(cluster_indices{long_unique_track_indices(i)}),...

        X(cluster_indices{long_unique_track_indices(i)}),...

        Y(cluster_indices{long_unique_track_indices(i)})];
```

```
end

% Add the tracks:

ma = ma.addAll(tracks);

% Compute MSD

ma = ma.computeMSD;

% % % Plot ALL MSD:

figure

ma.plotMSD;

% % % Plot Ensemble Averaged MSD: 241016_20_10

cla

ma.plotMeanMSD(gca, true)

% % % Add error bars and zoom in:

mmsd = ma.getMeanMSD;
```

```
t = mmsd(:,1);

x = mmsd(:,2);

dx = mmsd(:,3) ./ sqrt(mmsd(:,4));

errorbar(t, x, dx, 'k')

xlim([0 1000])

ylim  ([0 5E7])


clear mean_singlestep_jump

mean_singlestep_jump(length(long_unique_track_indices)) = 0;

for i = 1:length(long_unique_track_indices)

   x_move = diff(X(cluster_indices{long_unique_track_indices(i)}));

   y_move = diff(Y(cluster_indices{long_unique_track_indices(i)}));

   mean_singlestep_jump(i) = mean(sqrt(x_move.^2 + y_move.^2));

end


more_mobile_indices = long_unique_track_indices(mean_singlestep_jump > 150); %Find all indices for long+unique tracks that are also mobile
```

4.2. Sorting algorithm

```
clear all

overlap_threshold = 500; % How far localizations in each frame can be apart from each other and still be considered from the same molecule (nm)

intensity_threshold = [300 260 350]; %input empirically derived intensity thresholds for each channel. (Blue green red)


%Select .csv image you want to work up:

[file{1},folder] = uigetfile('*.csv*','select a csv file to analyze','multiselect','off');

[file{2},folder] = uigetfile('*.csv*','select a csv file to analyze','multiselect','off');

[file{3},folder] = uigetfile('*.csv*','select a csv file to analyze','multiselect','off');


% This section determines which .csv files loaded correspond to each channel. NOTE: In order for this section to work properly, the files should follow a naming convention with the end of the file containing the dye (e.g. filename_AF488.csv)

filename{1} = file{1}(end-8:end);

filename{2} = file{2}(end-8:end);

filename{3} = file{3}(end-8:end);

for i = 1:3 %In this work we used 3 channels, so i = 1:3
```

```
    if filename{i}(1) == ' ';
        filename{i}(end-3:end) = [];
        filename{i}(1) = [];
    else
        filename{i}(end-3:end) = [];
    end
end
%  The variable 'file' can be used to reference the name of all the files you selected. If you
%  want to know which group is which file just type the variable “file” into the terminal to
% double check your input


% This section determines number of files selected and define them all as type cell so
downstream syntax works:
if iscell(file)
    nfile = length(file); %count number of files
else
    nfile = 1; %if only one file format is different (saved as string vs cell)
    file = {file}; %convert to cell
```

```
end


% This section pulls out x, y, and intensity info (Z) from csv files:

for i = 1:nfile

    [X Y Z] = csvimport([folder,file{i}], 'columns', {'"x [nm]"', '"y [nm]"', '"intensity
[photon]"'},'ignoreWSpace',true);


% This section removes any points which do not meet intensity_threshold for each channel:

    low_intensity_indices{i} = find(Z<intensity_threshold(i));

    X(low_intensity_indices{i}) = [];

    Y(low_intensity_indices{i}) = [];

    Z(low_intensity_indices{i}) = [];


% This section simply counts the number of localizations

    total_localizations(i) = length(X);

    if total_localizations(i) == 0

        intensities{i} = 0;

        localizations{i} = [1E12*i 1E12*i];

    else
```

```
        intensities{i} = Z;

        localizations{i} = [X,Y];

    end

end


% Pre-write matrices used for analysis:

min_distance(nfile,nfile,max(total_localizations)) = 0; %Shortest distance between point in set 1
with ALL points in set 2

min_distance_i = ones(nfile,nfile,max(total_localizations)).*-1; %Corresponding point in set 2

min_distance_boolean(nfile,nfile,max(total_localizations)) = false; %Shortest distance >
distance threshold


% This section finds localizations in each channel and pairs them based on whether they are
within a certain distance of each other (defined as overlap_threshold):

for i = 1:nfile

    for j = i+1:nfile

        for k = 1:size(localizations{i},1)

            distances = sqrt((localizations{i}(k,1)-localizations{j}(:,1)).^2 + ...

                (localizations{i}(k,2)-localizations{j}(:,2)).^2);
```

```
            [min_distance(i,j,k),min_distance_i(i,j,k)] = min(distances);

            if min_distance(i,j,k) < overlap_threshold

                min_distance_boolean(i,j,k) = true;

            else

                min_distance_i(i,j,k) = -1;

            end

        end

    end

end

toofar_indices = find(min_distance_i == -1);


[g3_index, g1_index, g2_index] = intersect(min_distance_i(1,3,:),min_distance_i(2,3,:));


% Any points that are too far away are automatically defined as an index of -1, so remove these
points (should all be grouped into the first point).

if g3_index(1) == -1

    g1_index(1) = [];

    g2_index(1) = [];

    g3_index(1) = [];
```

```
end

% This section finds which localizations are paired with localizations in ALL other channels (e.g. this molecule exists in both the red, green, and blue) using output from the previous section.

group_123_indices = [g1_index,g2_index,g3_index];

group_123_positions =
[localizations{1}(g1_index,:),localizations{2}(g2_index,:),localizations{3}(g3_index,:)];

group_123_intensities =
[intensities{1}(g1_index,:),intensities{2}(g2_index,:),intensities{3}(g3_index,:)];

% Now that these indices are known to be in all three channels, remove them from the group:

localizations{1}(g1_index,:) = [];

localizations{2}(g2_index,:) = [];

localizations{3}(g3_index,:) = [];

intensities{1}(g1_index,:) = [];

intensities{2}(g2_index,:) = [];

intensities{3}(g3_index,:) = [];

% Find indices for all points that are NOT in all three channels (will use this in further analysis)

remaining_localizations = total_localizations-length(g1_index);
```

```
% Now reanalyze the data with the 3-channel points having been removed in order to find points which are in TWO channels. NOTE: There is probably a cleaner analytical way to do this without rerunning the same analysis, but this is simpler and if it works it works.

% Pre-write matrices used for analysis:

clear min_distance min_distance_i min_distance_boolean

min_distance(nfile,nfile,max(remaining_localizations)) = 0; %Shortest distance between point in set 1 with ALL points in set 2

min_distance_i = ones(nfile,nfile,max(total_localizations)).*-1; %Corresponding point in set 2

min_distance_boolean(nfile,nfile,max(remaining_localizations)) = false; %Shortest distance > distance threshold


for i = 1:nfile

    for j = i+1:nfile

        for k = 1:size(localizations{i},1)

            distances = sqrt((localizations{i}(k,1)-localizations{j}(:,1)).^2 + ...

                (localizations{i}(k,2)-localizations{j}(:,2)).^2);

            [min_distance(i,j,k),min_distance_i(i,j,k)] = min(distances);

            if min_distance(i,j,k) < overlap_threshold

                min_distance_boolean(i,j,k) = true;
```

```
            else
                min_distance_i(i,j,k) = -1;
            end
        end
    end
end
toofar_indices = find(min_distance_i == -1);


% Now calculate all positions which overlap in channel 1 and 2 (1,2):
group_12_indices = find(min_distance_boolean(1,2,:));
if isempty(group_12_indices)
    group_12_indices = group_12_indices';
    group_12_positions =
[intensities{1}([],:),intensities{1}([],:),intensities{1}([],:),intensities{1}([],:)];
    group_12_intensities = [intensities{1}([],:),intensities{1}([],:)];
else
    group_12_positions =
[localizations{1}(group_12_indices,:),localizations{2}(min_distance_i(1,2,group_12_indices),:)]
;
```

```
    group_12_intensities =
[intensities{1}(group_12_indices),intensities{2}(min_distance_i(1,2,group_12_indices))];
end


% Repeat for (1,3)
group_13_indices = find(min_distance_boolean(1,3,:));
if isempty(group_13_indices)
    group_13_indices = group_13_indices';
    group_13_positions =
[intensities{1}([],:),intensities{1}([],:),intensities{1}([],:),intensities{1}([],:)];
    group_13_intensities = [intensities{1}([],:),intensities{1}([],:)];
else
    group_13_positions =
[localizations{1}(group_13_indices,:),localizations{3}(min_distance_i(1,3,group_13_indices),:)]
;
    group_13_intensities =
[intensities{1}(group_13_indices),intensities{3}(min_distance_i(1,3,group_13_indices))];
end
```

```
% Repeat for (2,3)

group_23_indices = find(min_distance_boolean(2,3,:));

if isempty(group_23_indices)

    group_23_indices = group_23_indices';

    group_23_positions =
[intensities{1}([],:),intensities{1}([],:),intensities{1}([],:),intensities{1}([],:)];

    group_23_intensities = [intensities{1}([],:),intensities{1}([],:)];

else

    group_23_positions =
[localizations{2}(group_23_indices,:),localizations{3}(min_distance_i(2,3,group_23_indices),:)]
;

    group_23_intensities =
[intensities{2}(group_23_indices),intensities{3}(min_distance_i(2,3,group_23_indices))];

end


% This section outputs the overlapping localizations into excel tables for Sarah to use in
downstream analysis

T_123 = table(group_123_positions(:,1),group_123_positions(:,2),group_123_intensities(:,1),...

    group_123_positions(:,3),group_123_positions(:,4),group_123_intensities(:,2),...
```

```
    group_123_positions(:,5),group_123_positions(:,6),group_123_intensities(:,3),...
    'VariableNames',{[filename{1},': x-positions'],[filename{1},': y-positions'],[filename{1},': intensities'],...
    [filename{2},': x-positions'],[filename{2},': y-positions'],[filename{2},': intensities'],...
    [filename{3},': x-positions'],[filename{3},': y-positions'],[filename{3},': intensities']});

T_12 = table(group_12_positions(:,1),group_12_positions(:,2),group_12_intensities(:,1),...
    group_12_positions(:,3),group_12_positions(:,4),group_12_intensities(:,2),...
    'VariableNames',{[filename{1},': x-positions'],[filename{1},': y-positions'],[filename{1},': intensities'],...
    [filename{2},': x-positions'],[filename{2},': y-positions'],[filename{2},': intensities']});

T_13 = table(group_13_positions(:,1),group_13_positions(:,2),group_13_intensities(:,1),...
    group_13_positions(:,3),group_13_positions(:,4),group_13_intensities(:,2),...
    'VariableNames',{[filename{1},': x-positions'],[filename{1},': y-positions'],[filename{1},': intensities'],...
    [filename{3},': x-positions'],[filename{3},': y-positions'],[filename{3},': intensities']});

T_23 = table(group_23_positions(:,1),group_23_positions(:,2),group_23_intensities(:,1),...
```

```
    group_23_positions(:,3),group_23_positions(:,4),group_23_intensities(:,2),...
    'VariableNames',{[filename{2},': x-positions'],[filename{2},': y-positions'],[filename{2},': intensities'],...
    [filename{3},': x-positions'],[filename{3},': y-positions'],[filename{3},': intensities']});


filename_output = 'grouped data test.xlsx';
writetable(T_123,filename_output,'Sheet',[filename{1},' + ',filename{2},' + ',filename{3}])
writetable(T_12,filename_output,'Sheet',[filename{1},' + ',filename{2}])
writetable(T_13,filename_output,'Sheet',[filename{1},' + ',filename{3}])
writetable(T_23,filename_output,'Sheet',[filename{2},' + ',filename{3}])


% This section outputs the individual localizations into excel tables for Sarah to use in downstream analysis (NOT paired localizations)
remove_g1_indices = unique([group_12_indices; group_13_indices]);
remove_g2_indices = unique([group_23_indices;permute(min_distance_i(1,2,group_12_indices),[3,1,2])]);
remove_g3_indices = unique([permute(min_distance_i(2,3,group_23_indices),[3,1,2]);permute(min_distance_i(1,3,group_13_indices),[3,1,2])]);
```

```
% Copy
g1_solo_positions = localizations{1};
g2_solo_positions = localizations{2};
g3_solo_positions = localizations{3};
g1_solo_intensities = intensities{1};
g2_solo_intensities = intensities{2};
g3_solo_intensities = intensities{3};

% Remove
g1_solo_positions(remove_g1_indices,:) = [];
g2_solo_positions(remove_g2_indices,:) = [];
g3_solo_positions(remove_g3_indices,:) = [];
g1_solo_intensities(remove_g1_indices,:) = [];
g2_solo_intensities(remove_g2_indices,:) = [];
g3_solo_intensities(remove_g3_indices,:) = [];

% Tabulate:
```

```
T_1 = table(g1_solo_positions(:,1),g1_solo_positions(:,2),g1_solo_intensities,...
    'VariableNames',{[filename{1},': x-positions'],[filename{1},': y-positions'],[filename{1},':
intensities']});
T_2 = table(g2_solo_positions(:,1),g2_solo_positions(:,2),g2_solo_intensities,...
   'VariableNames',{[filename{2},': x-positions'],[filename{2},': y-positions'],[filename{2},':
intensities']});
T_3 = table(g3_solo_positions(:,1),g3_solo_positions(:,2),g3_solo_intensities,...
    'VariableNames',{[filename{3},': x-positions'],[filename{3},': y-positions'],[filename{3},':
intensities']});


% Write to xlsm:
writetable(T_1,filename_output,'Sheet',filename{1})
writetable(T_2,filename_output,'Sheet',filename{2})
writetable(T_3,filename_output,'Sheet',filename{3})
```

5. References

(1) Hu, Y.; Ma, V. P. Y.; Ma, R.; Chen, W.; Duan, Y.; Glazier, R.; Petrich, B. G.; Li, R.; Salaita, K. DNA-Based Microparticle Tension Sensors (MTS) for Measuring Cell Mechanics in Non-Planar Geometries and for High-Throughput Quantification. *Angewandte Chemie International Edition* **2021**, *60* (33), 18044–18050.

(2) Bush, J.; Singh, S.; Vargas, M.; Oktay, E.; Hu, C. H.; Veneziano, R. Synthesis of DNA Origami Scaffolds: Current and Emerging Strategies. *Molecules 2020, Vol. 25, Page 3386* **2020**, *25* (15), 3386.

(3) Hu, Y.; Rogers, J.; Duan, Y.; Velusamy, A.; Narum, S.; Al Abdullatif, S.; Salaita, K. Quantifying T Cell Receptor Mechanics at Membrane Junctions Using DNA Origami Tension Sensors. *Nature Nanotechnology 2024* **2024**, 1–12.

(4) Glazier, R.; Brockman, J. M.; Bartle, E.; Mattheyses, A. L.; Destaing, O.; Salaita, K. DNA Mechanotechnology Reveals That Integrin Receptors Apply PN Forces in Podosomes on Fluid Substrates. *Nature Communications 2019 10:1* **2019**, *10* (1), 1–13.

(5) Ovesný, M.; Křížek, P.; Borkovec, J.; Švindrych, Z.; Hagen, G. M. ThunderSTORM: A Comprehensive ImageJ Plug-in for PALM and STORM Data Analysis and Super-Resolution Imaging. *Bioinformatics* **2014**, *30* (16), 2389–2390.

(6) Lambert, T. J. FPbase: A Community-Editable Fluorescent Protein Database. *Nature Methods 2019 16:4* **2019**, *16* (4), 277–278.

(7) Nishimura, T.; Tate, N.; Nakagawa, M.; Shimomura, S.; Shirasaka, S.; Miyata, Y.; Suzuki, H.; Tanida, J. Spatiotemporal Model for FRET Networks with Multiple Donors and Acceptors: Multicomponent Exponential Decay Derived from the Master Equation. *JOSA B, Vol. 38, Issue 2, pp. 294-299* **2021**, *38* (2), 294–299.

(8) Massey, M.; Kim, H.; Conroy, E. M.; Algar, W. R. Expanded Quantum Dot-Based Concentric Förster Resonance Energy Transfer: Adding and Characterizing Energy-Transfer Pathways for Triply Multiplexed Biosensing. *Journal of Physical Chemistry C* **2017**, *121* (24), 13345–13356.

(9) Bunt, G.; Wouters, F. S. FRET from Single to Multiplexed Signaling Events. *Biophys Rev* **2017**, *9* (2), 119.

(10) Fábián, Á. I.; Rente, T.; SzölloSi, J.; Matyus, L.; Jenei, A. Strength in Numbers: Effects of Acceptor Abundance on FRET Efficiency. *ChemPhysChem* **2010**, *11* (17), 3713–3721.

(11) OpenAI. ChatGPT . OpenAI: San Francisco 2025.

(12) *ATTO-TEC GmbH - ATTO 643*. https://www.atto-tec.com/ATTO-643.html?language=en (accessed 2025-03-21).

(13) *GitHub - tinevez/msdanalyzer: A MATLAB class for Mean Square Displacement analysis.* https://github.com/tinevez/msdanalyzer (accessed 2025-08-19).
